# Harmonized and Open Energy Dataset for Modeling a Highly Renewable Brazilian Power System

**DOI:** 10.1038/s41597-023-01992-9

**Published:** 2023-02-22

**Authors:** Ying Deng, Karl-Kiên Cao, Wenxuan Hu, Ronald Stegen, Kai von Krbek, Rafael Soria, Pedro Rua Rodriguez Rochedo, Patrick Jochem

**Affiliations:** 1 German Aerospace Center (DLR), Institute of Networked Energy Systems, Curiestr. 4, 70563 Stuttgart, Germany; 2 Department of Mechanical Engineering, Universidad San Francisco de Quito, Diego de Robles y Vía Interoceánica, Campus Cumbayá, 170901 Quito, Ecuador; 3 Energy Planning Program, Graduate School of Engineering (COPPE), Universidade Federal do Rio de Janeiro, Centro de Tecnologia, Bloco C, Sala 211, Cidade Universitaria, Ilha do Fundão, 21941-972 Rio de Janeiro, Brazil

**Keywords:** Energy modelling, Energy policy, Energy management

## Abstract

Improvements in modelling energy systems of populous emerging economies are highly decisive for a successful global energy transition. The models used–increasingly open source–still need more appropriate open data. As an illustrative example, we take the Brazilian energy system, which has great potential for renewable energy resources but still relies heavily on fossil fuels. We provide a comprehensive open dataset for scenario analyses, which can be directly used with the popular open energy system model PyPSA and other modelling frameworks. It includes three categories: (1) time series data of variable renewable potentials, electricity load profiles, inflows for the hydropower plants, and cross-border electricity exchanges; (2) geospatial data on the administrative division of the Brazilian federal states; (3) tabular data, which contains power plant data with installed and planned generation capacities, aggregated grid network topology, biomass thermal plant potential, as well as scenarios of energy demand. Our dataset could enable further global or country-specific energy system studies based on open data relevant to decarbonizing Brazil’s energy system.

## Background & Summary

The decarbonization of energy systems in developing countries, especially in the most populous ones, becomes a determinant factor for a global “well below 2 °C” target^1^. Achieving climate neutrality requires complete or nearly complete decarbonization of the electricity system. This goal is attainable today through many technologies that provide low-carbon or even carbon-free electricity–renewable energy, nuclear power, and fossil-fueled electricity with carbon capture and storage. Low social acceptance and low economic viability make the latter two technologies more challenging to deploy on a large scale, and their timely installation questionable. However, the generation profile and production costs of variable renewable energy sources (vRES) vary with the weather, i.e., the spatial location and the availability of wind resources and solar radiation. Consequently, the decision problems in the operation and planning of reliable, stable, and carbon-neutral power systems rely on large-scale models and datasets.

Open science promotes using open models to support the transition to carbon-neutral energy systems. Typically, such open models are populated with datasets specific to the power system. However, energy data can come from different sources, and the accessibility and licensing conditions of energy data affect the degree of openness of the modelling workflows^2^. For this reason, the open data can help drive and support the efforts of improving transparency and productivity^3^. In developed countries, especially in Europe, various energy system models are available as open source^4^. There are several platforms, for instance, the Open Energy Platform (https://openenergy-platform.org/) and Open Power System Data platform^5^, which coordinate various open datasets (such as climate, demand profiles, transmission grids, and scenarios) for modelling the European power system.

In contrast, energy system models for developing countries use opaque and, in most cases, inaccessible datasets. Using those datasets makes it difficult for global energy models to represent emerging nations accurately. Language barriers may further hinder researchers who belong to a different language region from utilizing available energy data.

As one of the five most populous countries, Brazil is a developing country with significant wind resources and solar radiation potential, albeit in the early stages of deployment. Brazil’s energy system is facing a strategic transition, and the rainforest constrains its capacity expansion. All this makes it valuable to understand the Brazilian energy system in detail and its potential contribution to the global energy transition. An important dataset for modelling the Brazilian energy system is published in the context of Brazil’s National Ten-Year Expansion Plan^6^. It contains the input data for the corresponding investment model^7^. However, modellers, who would like to use this dataset, must have Portuguese language skills and modelling experience. The latter is necessary, e.g., to understand the context behind certain abbreviations or numerical values, which may be either based on empirical data or generically made up to fill data gaps. In particular, the dataset is provided for four electric zones plus ten nodes, which limits analyses at higher spatial resolutions, for instance, on the federal state level.

In this context, our contribution is to make the existing energy data of Brazil better applicable for energy systems modelling. By providing the first publicly available, spatially explicit, harmonized, and English version of Brazil’s energy data, we enable researchers to replicate the Brazilian energy system and/or to improve the integration into global energy models starting from a common basis.

The assembled dataset comprises the following subcategories as detailed in the Methods: (i) geospatial data for Brazil, (ii) aggregated grid network topology, (iii) vRES potentials–profile and installable generation capacity, (iv) geographically installable capacity of biomass thermal plants, (v) hydropower plants inflow, (vi) existing and planned power generators with their capacity, (vii) electricity load profile, (viii) scenarios of sectoral energy demand and (ix) cross-border electricity exchanges. This dataset is resolved geographically by Brazilian federal states, and time series data are resolved by hours, spanning 2012–2020.

In this way, the presented dataset provides the essential information and foundation for the operational and expansion planning studies necessary to explore Brazil’s highly decarbonized energy future. For example, the dataset was used in the PyPSA-Brazil model^8^ to assess the impact of transmission grid expansion in the Brazilian power system. The dataset published in this paper has been updated and includes more years of data than the version used^8^.

## Methods

This work aims to create consolidated open energy data for Brazil based on open and accessible original datasets.

Supplementary Table S1 summarizes the sources and licenses of the raw data used for each subcategory of the dataset in this paper. The following subsections elaborate on knowledge of energy data in the Brazilian context, how we obtain each dataset from its sources, and assumptions made in processing and constructing the datasets.

### Geospatial data for Brazil

Brazil has five macroeconomic regions, four electric regions, 27 federal levels (26 states and one federal district–Brasília), and 5572 municipalities.

**Fig. 1:**
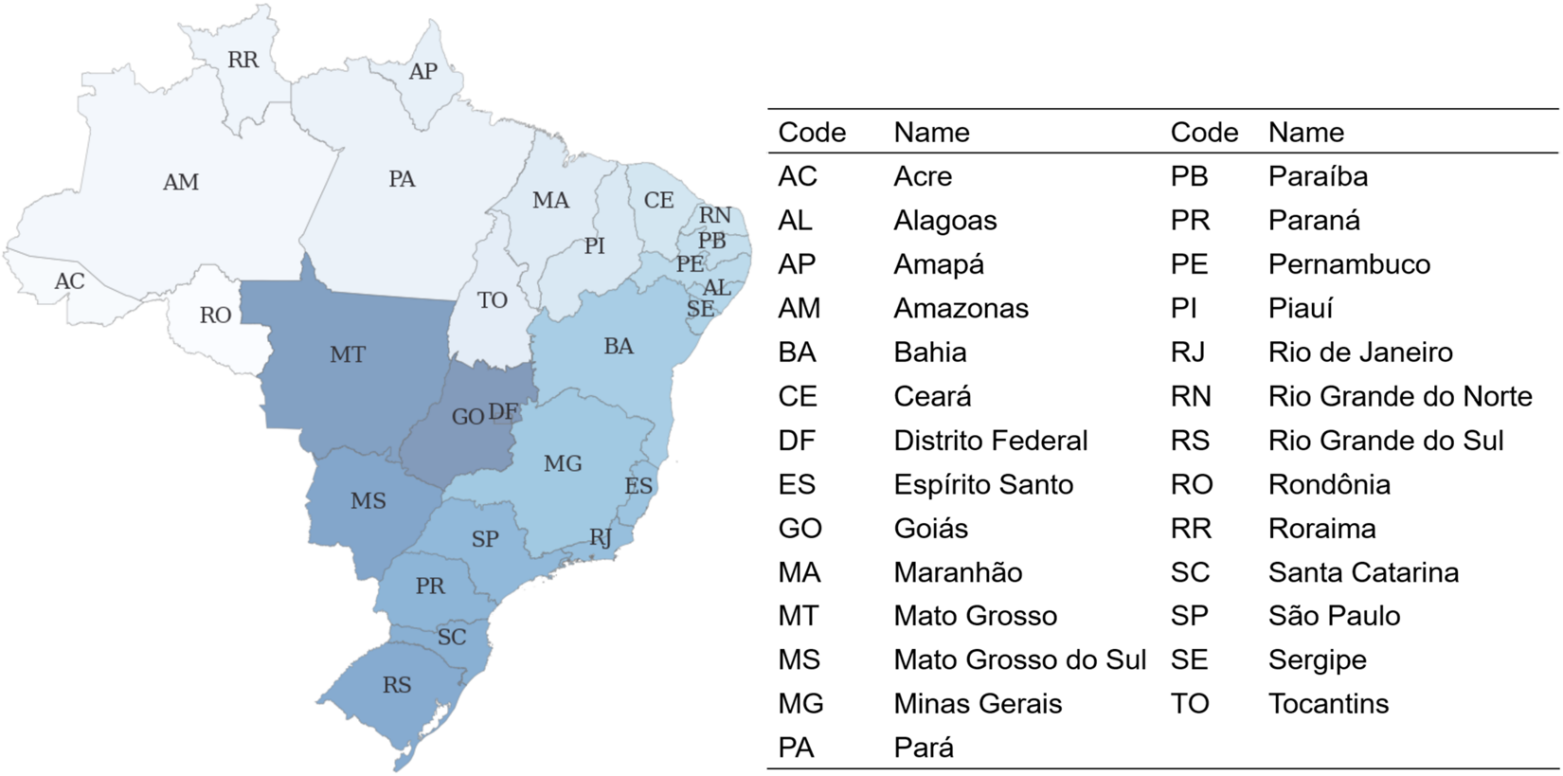
27 regions defined according to ISO 3166-2–Brazilian federal states–used in this study.

The spatial resolution of the dataset we provide is at ISO 3166-2 level^9^ and comprises 27 defined regions, i.e., federal level, illustrated in Fig. 1.

#### Data collection

Even though there are several map sources, the original dataset used is from the Brazilian Institute of Geography and Statistics (Portuguese: Instituto Brasileiro de Geografia e Estatística, IBGE)^10^. This choice is not only motivated by the licensing but also because IBGE is Brazil’s official map source and is considered the most credible source for the country’s borders and topography. The shapefile’s Coordinate Reference System (CRS) is SIRGAS 2000 (commonly known as EPSG:4674).

#### Data processing

These attributes in the original dataset^10^ are converted to English, and the CRS is re-projected to EPSG:4087. Only the federation state and the geometric information of the polygon are retained. In addition, representative coordinates (x, y) of the federal states are added and are considered as the centroid of the state polygon.

### Aggregated grid network topology

The power grid connects all power generators and loads. In Brazil, the electricity grid is known as the National Interconnected Network (Portuguese: Sistema Interligado Nacional, SIN) and is managed by the National Electricity System Operator (Portuguese: Operador Nacional do Sistema Elétrico, ONS). ONS divides Brazil into four electric regions, including several federal states, as shown in Table 1. SIN has a total length of 167,000 km and connects almost the entire country (96.6% of the national territory), except for some isolated places in the northern region. Over the next few decades, 434 lines with a total length of 32,000 km are planned to be built^11^.

**Table 1:** Electrical regions defined in the SIN and the federal states covered.

Electric regions in SIN	Federal states
North (N)	Pará, Tocantins, Maranhão, Amapá, Amazonas, Roraima
Northeast (NE)	Piauí, Ceará, Rio Grande do Norte, Paraíba, Pernambuco, Alagoas, Sergipe, Bahia
Southeast/Midwest (SE)	Espírito Santo, Rio de Janeiro, Minas Gerais, São Paulo, Goiás, Distrito Federal, Mato Grosso, Mato Grosso do Sul, Acre, Rondônia
South (S)	Rio Grande do Sul, Santa Catarina, Paraná

#### Data collection

Energy Research Office (Portuguese: Empresa de Pesquisa Energética, EPE) is a state-owned organization in Brazil that conducts studies and research to provide technical support for outlining medium- and long-term energy planning in Brazil for the design and implementation of national energy policy. EPE identifies potential energy sources for national development and contributes to research for auctions in the energy sector.

The complete grid topology of Brazil is taken from the dataset published by the EPE, called EPE Webmap^11^. The original datasets are in shapefiles, with transmission line data as the line layer and substation and generator data as the point layer. All lines, substations, and power plants have individual shapefiles, classified by their operational status–existing or planned–and, for power plants in particular, by their plant type. The CRS of the shapefile is SIRGAS 2000. The attributes are specified in Portuguese and include name, plant operator, voltage level, year of operation, and line length, among others. Substations, transmission lines, and different types of power plants shapefiles are used to derive the network topology. Supplementary Table S2 lists the number of records in the original datasets used.

#### Data processing

We provide the results of two aggregated networks–one for the existing network only and one for the existing and planned networks.

**Fig. 2:**
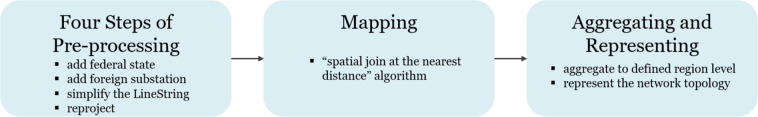
Overview of processing grid network data.

Each federal state is modelled as a node located in its geometric centre, connected by transmission lines in operation and in the National Ten-Year Plan^6^. We assume that existing and planned transmission lines are operating regardless of the scenario year, so we add up the transmission capacity and ignore the reference year. The original data does not provide information on the connection of the lines to substations or power plants; however, this is necessary to construct the grid topology. For this purpose, we use the heuristics method to connect the starting and ending points of transmission lines to nearby substations or power plants. The analysis has three parts: four steps of pre-processing, mapping, aggregating and representing, as displayed in Fig. 2. For geospatial analysis, we use the geopandas package in python.

Before the mapping action, there are four pre-processing steps to make the “spatial join at the closest distance” algorithm effective.

1. The federal states to which the substations and power plants belong are added to the attribute table according to their geographical locations.
2. Information on existing foreign substations connected to the SIN is added manually based on^6^. This is because the transmission lines indicated in the original line layer contain international connections, while information about substations outside Brazil is not specified. Added attributes include the name of the substation, the operator, the voltage, and the geometry. In addition, a new attribute, namely state, is added to identify the country to which it belongs using the ISO 3166-1 alpha-3 code. The state of the substation abroad is three characters, whereas, in Brazil, it is two characters. The geometry added manually is the longitude and latitude where the substation is located. An exception is the SE Macagua substation, located in Venezuela. Its actual location is (8.304, −62.668). However, it is designated as (4.530, −61.138). This is because, in the original data, the transmission line to the Boa Vista substation ends here. Additionally, the heuristic algorithm is based on the nearest distance criterion.
3. LineString in the transmission line layer has to be further processed by converting MultiLineString to LineString and closed LineString to open LineString.
4. The shapefiles are reprojected to EPSG:4087 so that the distance-based calculations are robust.

After pre-processing, we use the “sjoin_nearest” function of the Geopandas package in Python to map the start and end points of the line layer and the geometry of the substation and power plant. The maximum distance to query the nearest geometry starts from an initial distance of 1 km and increases by 1 km in each subsequent query. Table 2 reveals the statistics of the mapping results, where sub_0 represents the start point, and sub_1 represents the endpoint. More than 90% of the mappings (96.1% of the starting points and 94.4% of the ending points) are within 1 km. The line that causes the most significant deviation in the mapping is LT 230 kV Itapaci – Mineradora Maracá (the line name in the original data), with a length of 85 km, especially the mapping of its endpoints, since the nearest points of the line’s start and end points are the Itapaci substation.

**Table 2:** Statistical summary of the mapping–distance to sub_0 and to sub_1, km.

	to sub_0	to sub_1
count	2402	2402
mean	1.3	1.4
std	2	3
min	1	1
50%	1	1
90%	1	1
95%	1	2
max	4.8	8.2

Note: sub_0 is the starting substation, while sub_1 is the end substation in the mapping.

The final step is to aggregate these lines to represent the network topology between each federal state. Depending on the federal state information, only trans-state transmission is selected, which assumes that potential grid bottlenecks are not considered inside the federal states–copper-plates assumption^12^. The original dataset does not have information on whether the lines are alternating current (AC) or high-voltage direct current (HVDC) lines. There are several duplicate entities for HVDC lines, such as Porto Velho - Araraquara and Xingu - Estreito; these records are removed. The transfer capacity of the HVDC lines is supplemented manually with information from various sources, as specified in Table 3.

**Table 3:** Transfer capacity of HVDC lines added manually, MW.

Line name	Transfer capacity	Source
LT 600 kV Foz do Iguaçu – Ibiúna C1	3150	^62^
LT 600 kV Foz do Iguaçu – Ibiúna C2	3150	^62^
LT 600 kV Coletora Porto Velho – Araraquara, C1/C2	3150	^63^
LT 600 kV Coletora Porto Velho – Araraquara, C3/C4	3150	^63^
LT 230 kV Coletora Porto Velho – Porto Velho, C1	400	^63^
LT 230 kV Coletora Porto Velho – Porto Velho, C2	400	^63^
LT 800 kV CC Xingu – Estreito	4000	^64^
LT 800 kV CC Xingu – Terminal Rio	4000	^64^
LT 800 kV CC Graça Aranha – Silvânia	4000	^64^
LT 500 kV Rincón de Santa Maria – Garabi I C1	1100	^65^
LT 500 kV Rincón de Santa Maria – Garabi II C1	1100	^65^
LT 230 kV Livramento 2 – Rivera C1	70	^6^
LT 500 kV Candiota – Melo C1	500	^6^

The number of circuits in each transmission line is added to calculate the transfer capacity of AC lines. “C1” and “C2” in the line names represent the first circuit and the second circuit, marking each line of the parallel circuit, while “CD” indicates a double circuit^13^. Therefore, each line defaults to a single circuit, while lines with a “CD” tag in the line name are set to a double circuit. However, the original dataset had no information on the physical characteristics of the lines, such as the conductor resistance, inductance, and capacitance of each transmission line. Therefore, we assume that each line is four bundles of conductors. The remaining transmission lines of different voltage levels are unified as parallel lines of 380 kV, thus forming an equivalent transmission network. This enables the transmission capacities starting in the same federal state and ending in another identical federal state to be added. To calculate the transmission capacity of the equivalent transmission system, the lines are assumed to be three-phase overhead lines and of type 490-AL1/64-ST1A^14^.

The transfer capacity (apparent power) is calculated:

1 $$S=\sqrt{3}UI,$$

The transmission losses

2 $$f=1-\frac{3{{\rm{R}}}_{L}^{{\prime} }{I}^{2}n}{S},$$

where:

*S* = apparent power, MW

*U* = voltage level, kV

*I* = nominal current of the wire, kA

${R}_{L}^{{\prime} }$ =  DC resistance rating of the conductor at operating temperature for the wire, Ω/km

*n* =  number of bundle conductor, n = 4

*f* =  the transfer efficiency is considered as 1 minus the effective loss of each line.

**Fig. 3:**
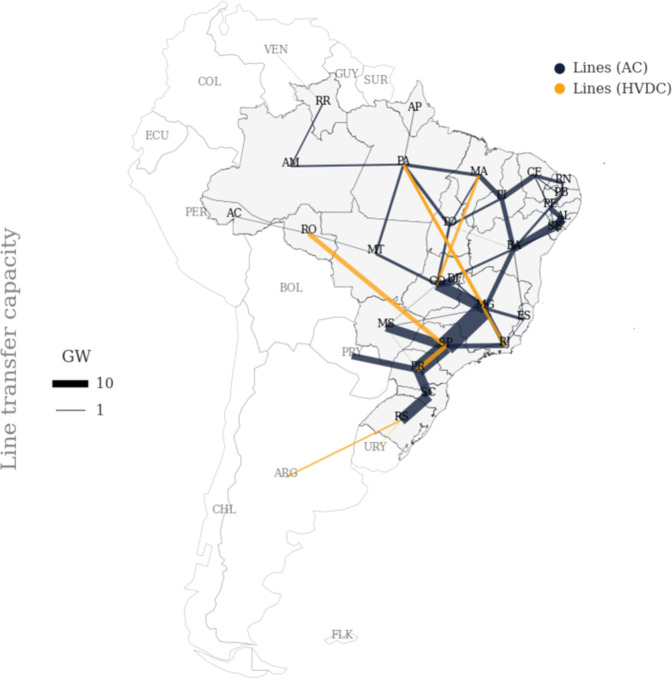
Transfer capacity between the defined regions, GW.

In aggregation, transmission capacity is accumulated, and efficiency and line length are averaged out. In Fig. 3, the results are illustrated.

In this paper, we focus on the data derived from the original data of the transmission network. We know that it is also helpful to analyze distribution network data at the regional level. However, to the best of our knowledge, there is a lack of appropriate public data sources.

### Power plants

Generators are an integral part of the energy industry, responsible for producing electricity and injecting it into the grid–transmission and distribution–to reach consumers.

#### Data collection

There are several official generator databases in Brazil, for example, ANEEL-SIGA^15^ published by National Electric Energy Agency (Portuguese: Agência Nacional de Energia Elétrica, ANEEL), EPE Webmap^11^, ONS Historical Database^16^. ANEEL-SIGA is the Generation Information System and contains information on power plants from the granting phase to the decommissioning phase. EPE Webmap refers to the Geographic Information System of the Brazilian Energy Planning Studies. It is a geo-referenced database containing official information for Brazil’s medium and long-term energy planning. The power plants in the ONS Historical Database mainly refer to those which are operated by ONS and are part of its SIN. Generally, when a power plant is in operation, it implies that it is connected to the SIN. Some exceptions exist, such as isolated systems supplied by local generations and not connected to the SIN.

Power plants delegated by ANEEL have a single generation unit code–CEG (company identification code). Table 4 explains its format. All three datasets have CEG, renamed “plant_id” for clarity. The types of plants considered in the three datasets are different, as shown in Table 5. ONS defines the coarsest power plant types. However, ONS distinguishes hydroelectric power generation from hydropower and pump types, while neither ANEEL-SIGA nor EPE Webmap has information on pump types. To compare datasets differentiated by plant type, the installed capacities for generation are summed to the plant types defined by Harmonised in Table 5. In addition, both ANEEL-SIGA and EPE Webmap provide geographic coordinates, while the ONS Historical Database reveals only the electric regions and federal states in which the plant units are located. ANEEL-SIGA and EPE Webmap comprise the power units in operation and planned, while ONS only contains the power plants in operation.

**Table 4:** CEG definition.

GGG.FF.UF.999999-D	
Part	Explanation
GGG	generation Type
FF	the fuel type abbreviation
UF	federal state abbreviation
999999-D	unique number with identification digit

**Table 5:** The types of power plants used in the three datasets.

Harmonised	ONS Historical Database	EPE Webmap	ANEEL-SIGA
solar_pv	solar_pv	solar_pv	solar_pv
on_wind	on_wind	on_wind	on_wind
nuclear	nuclear	nuclear	nuclear
thermal	thermal	biomass_thermal	thermal
		fossil_thermal	
hydro	hydro	small_hydro	small_hydro
		mini_hydro	mini_hydro
	hydro_pump	hydro	hydro
			wave

Ideally, we should combine all three datasets to obtain a complete dataset. However, merging the three datasets into one is challenging because they have different granularities and do not complement each other. Since they are all official datasets, it is also challenging to determine which dataset is more reliable. Table 6 gives the statistics of the three datasets regarding the number of data entities, attributes, unique plant IDs and names, while Table 7 describes the total installed capacity for each plant type. Table 6 shows that the number of data entities is evidently different, in which ANEEL-SIGA covers the most data entities and attributes. In addition, only the ANEEL-SIGA dataset has a unique and complete identification of the plant IDs. Hence, the data entities of EPE Webmap and ONS Historical Database can be grouped compared to ANEEL-SIGA. Despite having an equal number of attributes, the ONS Historical Database distinguishes itself from ANEEL-SIGA by including specific details, such as names and IDS of power plant units that are only used by ONS,  which are less relevant for energy system analysis. In contrast to ANEEL-SIGA, the ONS Historical Database does not provide planned power plant units and geography information. ANEEL-SIGA covers almost all attributes provided by ONS Historical Database and EPE Webmap. Table 7 indicates that the total installed capacity of each type of plant in the EPE Webmap and ONS Historical Database is similar but contains less installed capacity of wind, PV and thermal plant types compared to ANEEL-SIGA. The difference between the three datasets may result from the following reasons:

1. EPE Webmap covers mainly centralised generation, whose operating mechanisms are self-generation and public utilities. In addition to the plants in the EPE Webmap, the ANEEL-SIGA database includes distributed generation under the net metering scheme and small-scale backup generators. ONS Historical Database contains the plants dispatched in SIN.
2. The dataset updates between ONS Historical Database, EPE Webmap, and the ANEEL-SIGA database are not synchronised. ONS publishes information on operating power plants on an annual basis–we use the latest data until December 2022. The latest update of the EPE Webmap was in September 2020. On the contrary, the ANEEL-SIGA database is constantly updated with the granting of power plants. However, the historical versions of ANEEL-SIGA are not accessible.
3. Different definitions of plant units. ANEEL documents each data entity of plant unit based on when they received their grant, while ONS defines projects based on their operating units.

**Table 6:** Statistical comparison of data entities between datasets–EPE Webmap, ANEEL-SIGA, and ONS Historical Database.

Number of	ONS Historical Database	EPE Webmap	ANEEL-SIGA
**data entities**	4191	3178	10541
**attributes**	15	11	15
**unique plants IDs**	1389	3160	10541
**unique plants names**	1388	2984	10283

**Table 7:** Comparison of installed capacity (GW) per plant type between datasets–EPE Webmap, ANEEL-SIGA, and ONS Historical Database.

Type	ANEEL-SIGA	EPE Webmap	ONS Historical Database
**hydro**	111.37	110.41	110.43
**nuclear**	3.34	3.40	1.99
**on_wind**	31.00	20.95	22.35
**solar_pv**	24.07	4.76	6.43
**thermal**	52.45	44.86	34.81

Note: the installed capacity is the sum of units operating in 2018; plant type is defined by Harmonised in Table 5.

As a result of the above discussion, we decide to use ANEEL-SIGA as the original input for several reasons: (1) available geographic coordinates, (2) it covers all relevant attributes for the energy system analysis of the other two datasets, (3) more data entities with the higher total installed capacity than the other two datasets, which includes operating and planned plant units,  (4) unique and complete identifier of the data entities–plant ID, and (5) continuously updated.

There are 10,541 power plant units with 21 attributes in ANEEL-SIGA. From the database, these attributes include the name of the power plant, the plant ID, operational status (The status are “operation”, “construction”, and “construction not started”, which is defined by the original dataset and is complete.), federal state to which it belongs (each entity can be a single power plant or a power plant unit consisting of multiple power plants, for example, a wind farm operating multiple wind turbines. The location of the power plant units provided determines the federal state.), city it belongs to, plant type, primary energy source, fuel type, installed capacity, geographic coordinates of each generator, production capacity, primary fuel type, time in operation, and phase-out time. The CRS used for the ANEEL-SIGA dataset is SIRGAS 2000, with coordinates expressed in degrees minutes seconds (DMS).

We match the power plants based on the plant IDs to provide insight into the consistency of ANEEL-SIGA compared to EPE Webmap and ONS Historical Database. Before matching, we group capacity, federal state, plant name, plant type and operation status based on plant ID. For details, see compare_power_plant_source/results/installed_capacity _comparison.xlsx of the GitLab project^17^. ANEEL-SIGA and EPE Webmap have 3035 data entities with the same plant ID, while EPE Webmap has 130 data entities not included in ANEEL-SIGA and 7512 data entities from ANEEL-SIGA do not appear in EPE Webmap. However, even with matching plant IDs, the installed capacity (300 entities), federal state information (77 entities), and the name of the plant unit (277 entities) may differ. The comparison between ANEEL-SIGA and ONS Historical Database shows that 1330 data entities match based on plant IDs. ONS Historical Database has 71 data entities with unique plant IDs, while ANEEL-SIGA has 9216. There may be discrepancies in the installed capacity (292 entries), federal state information (36 entities), and plant unit plan (132 entities) when plant IDs match.

#### Data processing

Even though the ANEEL-SIGA data can be displayed online through PowerBI, it only provides a download link. There are slight inconsistencies between the downloaded files (in XLSX format), for instance, plant coordinates and plant names. Therefore, the dataset provided in this paper is based on the version downloaded by the authors on June 9, 2021.

The ANEEL-SIGA data is constantly updated. The coordinates of the power plants need to be added to ensure completeness, and they should fall within the Brazilian range. Since the entity of city names is complete, we assign the missing coordinates of the plant with the city’s location. An individual power plant unit in more than one city can have multiple values in the “city” property. For those plant units, only the first value of the city name is considered. There are 847 entities with missing coordinates or coordinates outside Brazil. Once the coordinates have all been replenished, information on the federal states is updated with the coordinates.

The installed capacity of each power plant unit determines its size. In the original dataset, the capacity is given in kilowatts and provided separately for granting, regulation and inspection purposes. The granted capacity is the capacity considered in the act of granting, whereas the regulated capacity corresponds to the capacity considered from the commercial operation of the first generating unit. The actual guaranteed power is, on the other hand, represents the average actual production. Given that information on the regulation capacity may not be available for all power plant units, the granted capacity is deemed a suitable representation of the installed capacity. In addition, the units of installed capacity are converted to megawatts.

**Fig. 4:**
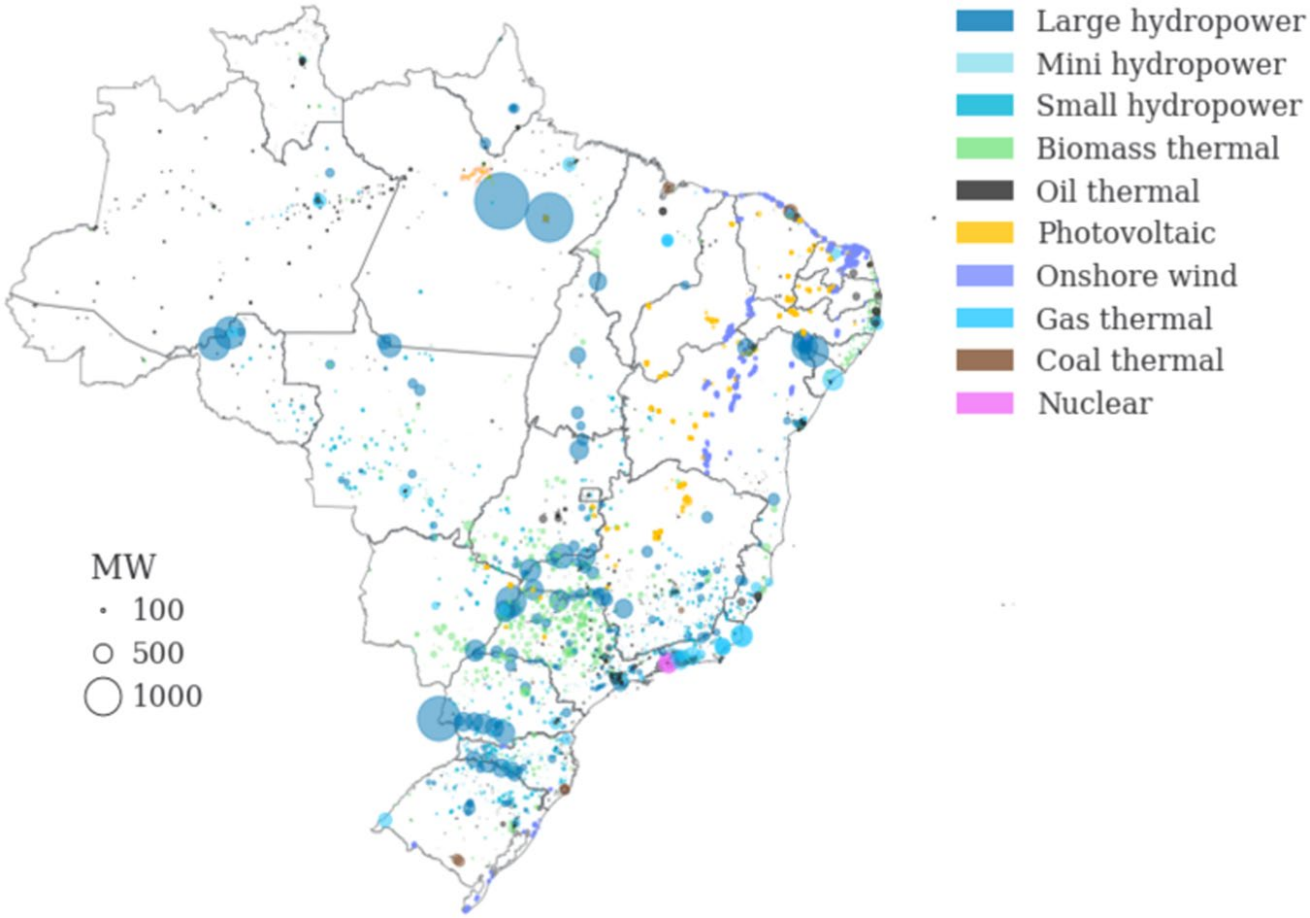
Existing and planned power plant capacity.

The information on the types of power plants in the original dataset is divided into eight types, summarized in Table 8. The single wave power plant, Porto do Pecém, installed in the state of Ceará, with a power of 0.05 MW, is classified in this paper as a small hydropower type. Depending on the properties of the fuel source, thermal power plants are subdivided into oil-fired, natural gas-fired, coal-fired, and biomass-fired. In total, therefore, there are ten generic types of plants. Figure 4 illustrates the results of power plant distribution.

**Table 8:** Power plants description and their abbreviations.

Short name in our dataset	Full name	Abbreviation (in Portuguese)	Explanation
hydro	Large hydropower plant	UHE	The hydropower plant with a capacity greater than 5MW and less than 50MW without those identified as small hydro
small_hydro	Small hydropower plant	PCH	The hydropower plant with a capacity greater than 5MW and less than or equal to 30MW with a reservoir area of up to 13 km^2^
mini_hydro	Mini hydropower plant	CGH	The hydropower plant with a capacity of 5MW or less
wave	Wave power plant	CGU	The energy comes from the water dynamics obtained from the sea waves. The energy comes from the kinetic energy of water from ocean waves. There is only one and the first wave power plant in Latin America, Porto do Pecém with 0.05MW in Ceará.
biomass_thermal, fossil_thermal	Thermoelectric plant	UTE	Generating energy with electricity released from any product that generates heat, such as bagasse from various plants, wood chips, fuel oil, diesel, natural gas, enriched uranium, and natural coal.
nuclear	Thermonuclear plants	UTN	Thermoelectric power plants, using the energy released by nuclear fission of uranium as a source
on_wind	Wind power plant	EOL	Converting the kinetic energy of the wind into electrical energy. So far, EOL refers to onshore wind power plants. Note: the short name–off_wind–refers to an offshore wind farm, which is not yet in place and does not have an official abbreviation.
solar_pv	PV power plant	UFV	Converting the sun’s energy into electricity through the photovoltaic effect, a voltage or corresponding current produced by a material when exposed to light.

Most entities have incomplete dates of commissioning and decommissioning. According to^18^, the missing date information indicates that the plants are active. We set the decommissioning date for those entities showing the same commissioning date and decommissioning date to be missing. Finally, the reference year is added Table 9.

**Table 9:** Comparison of annual electricity consumption (TWh) between ONS and EPE datasets.

Year	ONS	EPE	Δ = ONS−EPE
2012	511.7	448.1	63.6
2013	514.7	463.1	51.5
2014	539.5	474.8	64.7
2015	537.6	465.7	71.9
2016	541.5	461.8	79.7
2017	549.1	467.2	82.0
2018	554.3	474.8	79.5
2019	565.7	482.2	83.5
2020	557.2	475.6	81.5

In the post-processing, the installed capacity of the power plants is aggregated by federal state for each reference year according to the type and operational status of the power plant. This aggregation encompasses capacities derived from public service, self-generating, or independent production. The installed capacity of the reference year is determined under the assumption that the operational status of the plants is operational and that the commission time precedes the base year or is not specified. Records in the original dataset that pertain to the power plants with an operational status of “construction” or “not started construction” operational status are reclassified as “planning”. Finally, the values are accumulated according to the federal state, plant type, and operational status. The installed capacity differs based on the specified reference year, while the planned capacity is the same across all reference years. This is because 68.8% of the entities lack a decommissioning date, and as such, the decommission information is disregarded. As a result, we present the installed and planned capacity (GW) for each reference year under the project folder^17^ power_plants/resource/REFERENCE_YEAR/aneel_installed_cap_per_state_operation_GW_REFERENCE_YEAR.xlsx.

We intend to exclude the economical parameters of the generating units. Although reference^4^ provides cost assumptions for each power plant based on the fuel type, we remain skeptical about the applicability of incorporating these cost assumptions into the scenario study. Harmonizing cost assumptions for generators is a complex task due to the wide range of cost estimates across different sources for each generator technology. In addition, the base year, scenario year, and technology horizon significantly impact the cost assumptions in the scenario analysis. For example, reference^7^ gives the cost assumptions for the scenario year 2029, which is used for the Brazilian National Ten-Year Energy Research Study.

Furthermore, when comparing our dataset with the European dataset of power plants provided by Powerplantmatching^19^, we identify several gaps in information, particularly with regards to the dates of installation, retrofitting and decommissioning, the type of each hydroelectric plant and relevant technical parameters such as volume, dam height and storage capacity.

### Installable capacity for biomass thermal plants

Biomass can be burnt directly for heating or power generation, or converted into oil or natural gas substitutes. In the last 15 years, the generation of electricity from biomass thermal plants in Brazil has been increasing, from 6 GW to 14 GW, accounting for 13% of the capacity matrix of electricity for 2020. Sugarcane bagasse is the primary source of biomass.

#### Data collection

To the knowledge of the authors, there are no studies have specifically investigated the energy production potential of biomass thermal plants in Brazil. However, reference^20^ addresses the geographically installable capacity. In that paper, the authors estimate the potential for installable capacity for agricultural and agro-industrial residues where it is technically, environmentally sustainable and economically feasible. The theoretical capacity defines the maximum available bioenergy, subject to biophysical and agroecological conditions that hold down the growth of crops and residues, such as temperature, solar radiation, rainfall, and soil properties. This potential is limited by environmental constraints, as agricultural residues are critical biome regulators. As for the environmentally sustainable potential, the authors apply a theoretical constraint for removing residues to ensure environmental sustainability, such as preventing soil erosion and maintaining nutrient recycling. On the other hand, techno-economic viability refers to the fraction of the environmentally sustainable potential available under technological possibilities and logistic restrictions. It considers the competition of other non-energy uses of residues. As a result, only biomass residues spread within a 50 km radius from the power substations are economically feasible to be used in centralised power plants based on direct combustion of biomass in a Rankine power plant with an average efficiency of 18%. According to their assessment, the total economic potential in Brazil is 39 TWh/yr. The authors, with their permission, have generously provided us with the results of their paper’s economic potential in MWh/yr, which are spatially resolved at the municipal level.

#### Data processing

The primary energy source used in today’s biomass thermal plants is sugarcane bagasse, which is the dry pulpy substance remaining after grinding sugarcane to extract their juice. The contribution of residues is relatively small and thus negligible. Therefore, only the economic potential of biomass from residues is considered as additional installable capacity beyond the already existing and planned installations.

As a first step, we convert the potential production into the additional installable capacity by assuming an annual availability factor of biomass at 0.6^21^. Then the values are aggregated at the federal-state level.

The biomass thermal plants included in the study^21^ are obtained from centralized plants published by ANEEL, which are no longer accessible. We assume that the geographic distribution of the biomass thermal plants they considered is similar to that covered in the Subsection of Power plants. Although the number of hours in a year depends on whether there is a leap year, we have assumed in our calculations a constant number of 8760 hours per year. This allows us to calculate the geographically installable capacity for each state as follows:

3 $${C}_{i}=\sum _{i}\left(\frac{PR}{f\cdot 8760},+,C,{I}_{i},+,C,{P}_{i}\right),$$

where:

*C* = geographically installable capacity, MW

*i* = the federal state

*PR* = the residual potentials at municipality level, MWh

*f* = annual availability factor

*CI* = installed capacity of biomass thermal plants, MW

*CP* = planning capacity of biomass thermal plants, MW.

Since the installed capacity differs for each reference year, the geographically available installed capacity varies accordingly. Therefore, we provide data for each reference year to illustrate the changes.

### Electricity load profiles

Future energy systems are likely shift to renewable electricity as the primary energy source. As a result, the temporal distribution of energy consumption becomes increasingly relevant in the design of future energy systems as the share of vRES increases and consumption patterns change. At the same time, the spatial distribution of energy consumption gains importance as the generation and consumption of renewable energy become asynchronous across regions.

#### Data collection

EPE conducts studies and projections of the consumption and load of electricity in the Brazilian electric sector by obtaining historical data and projections from distribution agents, self-producers, and free consumers^22^. In comparison, the ONS reports up until 02/03/2021 the load of the national electricity system and the generation of power plants supervised by the ONS. Since then, ONS has reported the global load, which includes the generation of unsupervised plants and is unrelated to the ONS^23^.

ONS publishes hourly load profiles for its four electric regions in SIN^24^, while EPE provides annual sectoral electricity consumption or consumers for each federal state^25^. Table 1 indicates each electric region and the federal states it contains. ONS’s hourly profile covers the period of 1999-2020, while the EPE dataset ranges from 2012 to 2022 (retrieved in April 2021). However, the value of total power consumption provided by ONS is greater than that of EPE, as seen in Table 9. The reasons for this difference are the physical losses in transmission and distribution and the physical representation in the SIN^26^. The differences between the ONS and EPE datasets are illustrated in Table 10, where the regional differences are depicted. In addition, the time zone of the time series data published by ONS is UTC-3–Brasília Time.

**Table 10:** Comparison of annual electricity consumption differences by electric region between ONS and EPE datasets.

Year	N	NE	S	SE
2012	−4%	17%	11%	14%
2013	7%	16%	10%	9%
2014	17%	14%	9%	12%
2015	20%	16%	10%	13%
2016	21%	17%	12%	14%
2017	22%	18%	12%	14%
2018	24%	19%	11%	13%
2019	25%	18%	11%	14%
2020	22%	19%	11%	13%

#### Data processing

Both the ONS dataset and the EPE dataset are used.

The ONS dataset includes an hourly time series for each of its four electric regions in the SIN, but it has one missing value per year per region, except for 2019 and 2020. The greatest number of missing values occurs in 2014 with 25, with no data available for 1st February 2014. To fill the missing values, we use the values from one week earlier. In addition, six values are harmful in the time series for the northern region. These values are trimmed to zero as this is a gross error.

We use the EPE dataset as an allocation factor to decompose the ONS load profiles at the Brazilian federal state level. Therefore, there are two allocation factors–annual consumption and annual consumers. This means that we assume that the seasonal, intraweekly, and intraday variations remain consistent across states belonging to the same electric region but differ in magnitude. The load profiles for each federal state contain the transmission and distribution losses endogenously. We assume that states belonging to the same electrical region have the same pattern and different magnitude loss curves. Our assumption may be conservative since we provide the losses for transmissions between federal states in the Subsection of Aggregated grid network topology. However, we expect to retain the distribution losses to represent them in the dataset we provided.

**Fig. 5:**
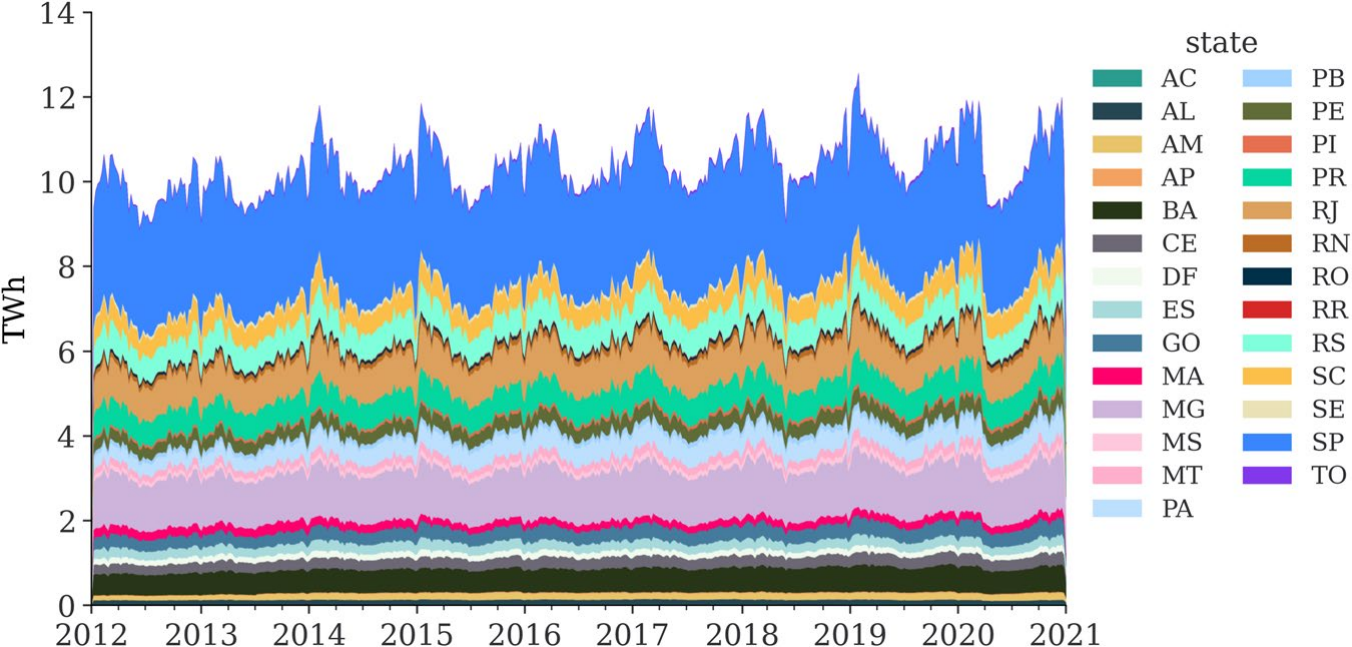
Electricity load at federal states (weekly) distributed by annual consumption. The dataset to be published is resolved hourly.

As the EPE allocation factors only apply for 2012–2020, the time horizon for electricity consumption in the federal states provided in this study applies only to 2012–2020. Figure 5 illustrates the results of the electricity load in the federal state, which is the sum of electricity consumption and the physical losses in the SIN.

### Scenarios of energy demand

Energy demand scenarios facilitate a strategic assessment of possible pathways for long-term planning and their respective internal consistency and associated uncertainties. Sector-specific modelling allows variations in demand from different resources and sectors to be estimated nationally. However, diverse models, methods, and assumptions lead to different scenarios and represent research positions–conservative or optimistic, dependent on fossil or renewable energy.

In energy system studies, future electricity demand can come from other studies or be calculated exogenously in the energy system model. Those energy studies, which use the future electricity demand exogenous, need to explain whether the electricity demand adopted from others takes into account mitigation measures consistent with Brazil’s first National Development Plan (2022 update), the Paris Agreement, or other mitigation targets. Otherwise, the studies cannot conclude the contribution of a given scenario to the mitigation targets, for instance,^27^. This makes it difficult to interpret their results, especially whether they are consistent with the Paris Agreement.

The updated first Brazilian nationally determined contribution (NDC) confirms the commitment to reduce its greenhouse gas emissions by 37% in 2025, compared to 2005. Additionally, Brazil pledges to reducing its emissions by 50% in 2030, compared to 2005, and aims to achieve climate neutrality by 2050 as its long-tern objective^28^. Brazil’s updated NDC is broad in scope, with economy-wide absolute targets. It takes into account means of implementing, undertaking mitigation, and adaptation actions in all economic sectors^28^. These targets would be translated into sectoral policies and measures to be detailed and implemented by the Brazilian federal government. These sectoral initiatives must be exogenously modelled to calculate the sectoral electricity consumption in each region. Only then can energy system models use the sectoral electricity consumption as input in energy system studies, allowing for a better understanding of their impact on power system operation and expansion.

Having a comprehensive understanding of sectoral energy demand published in reputable studies enables researchers in energy system modelling to accurately emulate demand-related parameterization and manage uncertainties.

#### Data collection

There are numerous scenarios for the future energy demand of Brazil. The most famous ones are published in three studies: (i) World Energy Outlook (WEO), (ii) EPE’s Long-term National Energy Plan (Portuguese: O Plano Nacional de Energia, PNE), and (iii) the exogenous energy demand studies by COPPE researchers.

The WEO scenario of the International Energy Agency (IEA) is considered the most authoritative source of insights into the world’s energy demand. It updates its sector demand scenarios annually, region by region. The latest WEO study for 2021^1^, regarded as “WEO2021” in this paper, provides reference data of historical demand for 2010, 2015, 2019, and 2020, as well as the sectoral energy demand scenarios for Brazil to 2050, with a five-year time span. The data for Brazil can be found in the extended CSV file in the WEO2021 study.

EPE’s PNE is a fundamental instrument for Brazil to outline the government’s strategy regarding the expansion of the energy sector in the coming decades. The latest plan,  PNE 2050^29^, was released in December 2020, and extends the horizon to 2050. PNE 2050 provides projections of sectoral demand every ten years (i.e., 2030, 2040, and 2050) depending on the economic and sector assumptions. Our comparison relies on the PNE 2050 study, referred to in this paper as “PNE2050”. However, the PNE2050 does not provide numerical data, instead presenting it as a table or charts for each end-use sector. We, therefore, have to extract these values manually and create a CSV file accordingly.

COPPE is the most prestigious research institute in Brazil that studies energy planning in Brazil and the world. We refer to their scenario studies as “COPPE”. Out of the 133 scenarios provided by COPPE, we selected three scenarios, as they are so far the latest and have distinct transition paths. COPPE scenarios have five-year time steps; however, the data we received only contain the years 2030, 2040, and 2050. Sectoral demand for 2010 or 2015 is the starting point for the scenario assumptions. In the following, the three COPPE scenarios are shortly described.

To enhance the transparency of energy scenarios^30^, this work creates a matrix of energy demand scenarios. This matrix (shown in Supplementary Tables S4-S6) provides a summary of the main criteria used by previous studies to model final energy consumption scenarios up to 2050 in Brazil, following the comparisons described in^31^. Trend scenarios are considered, which maintain a level of effort in climate action similar to current policies and NDCs, and ambitious mitigation scenarios aligned with the global goals until the end of the century on the stabilization of the average temperature increase of the planet relative to pre-industrial times by 2 °C and 1.5 °C. These scenarios highlight the role that electrification may play in the different sectors to achieve climate goals. However, the electrification of the transport sector in Brazil may not be as achievable as in other regions due to the critical role that traditional and advanced biofuels can play. This is especially evident in the lowBECCS scenario, which signifies a low role for bioenergy with carbon capture and storage.

#### Data processing

We first normalize the units of demand values for the three studies to PJ because they are different in the raw data, i.e., PJ for the WEO2021, Mtoe (million tonnes of oil equivalent) for PNE2050 and EJ for COPPE. After that, we give aliases in a format of XXXX_YYYY to represent the studies and the corresponding scenarios. For example, the alias COPPE_BAU represents the Business as Usual (BAU) scenario for the publication of the COPPE studies.

We align the end-use sectors and energy carriers in PNE2050 and COPPE with WEO2021 based on^1,29,32–34^, as the different definitions prevent comparisons between them. Supplementary Table S3 describes the correspondence. WEO2021 does not provide a value for the end-use sector named “Other”. We assume that the value for the end-use sector “Other” is the difference between total final consumption (TFC) and sectoral demand:

4 $${{\rm{Other}}}_{i}={{\rm{TFC}}}_{s}-{D}_{s,i},$$

where:

*Other*_*i*_ = the energy demand for the energy carrier *i* in the end-use sector of “Other”,

*TFCs* = total final consumption for end-use sector *s*,

*s* = end-use sector *s*, *s* ∈ {Transport, Industry, Buildings},

*i* = the energy carrier *i*, i ∈ {Total liquids, Total gases, Total solid fuels, Total},

*D*_*s, i*_ = the energy demand for the end-use sector *s* and the energy carrier *i*.

PNE2050 data provides the most granular energy carriers, followed by WEO2021, while the COPPE scenarios divide the energy carriers into “electricity”, “liquid”, “gas”, “solid”, and “hydrogen”. Supplementary Table S3 lists all energy carriers for PNE2050. The WEO2021 scenario dataset includes TFC, the total value of energy carriers by physical state, i.e., “total liquids”, “total gases”, “total solid fuels”, as well as some of the more subdivided energy carriers. For instance, “total liquids” consists of “oil products”, “liquid biofuels”, and “hydrogen-based liquid fuels”^1^. However, “liquid biofuels” are not provided. Although an energy carrier, “hydrogen”, is provided in the COPPE scenarios, all scenarios have zero values. Therefore, we leave the “hydrogen” out. Even when hydrogen as final energy is zero, there is a critical hydrogen production as an intermediate energy carrier, which is input to produce other final energy forms. This intermediate product is not reported.

**Fig. 6:**
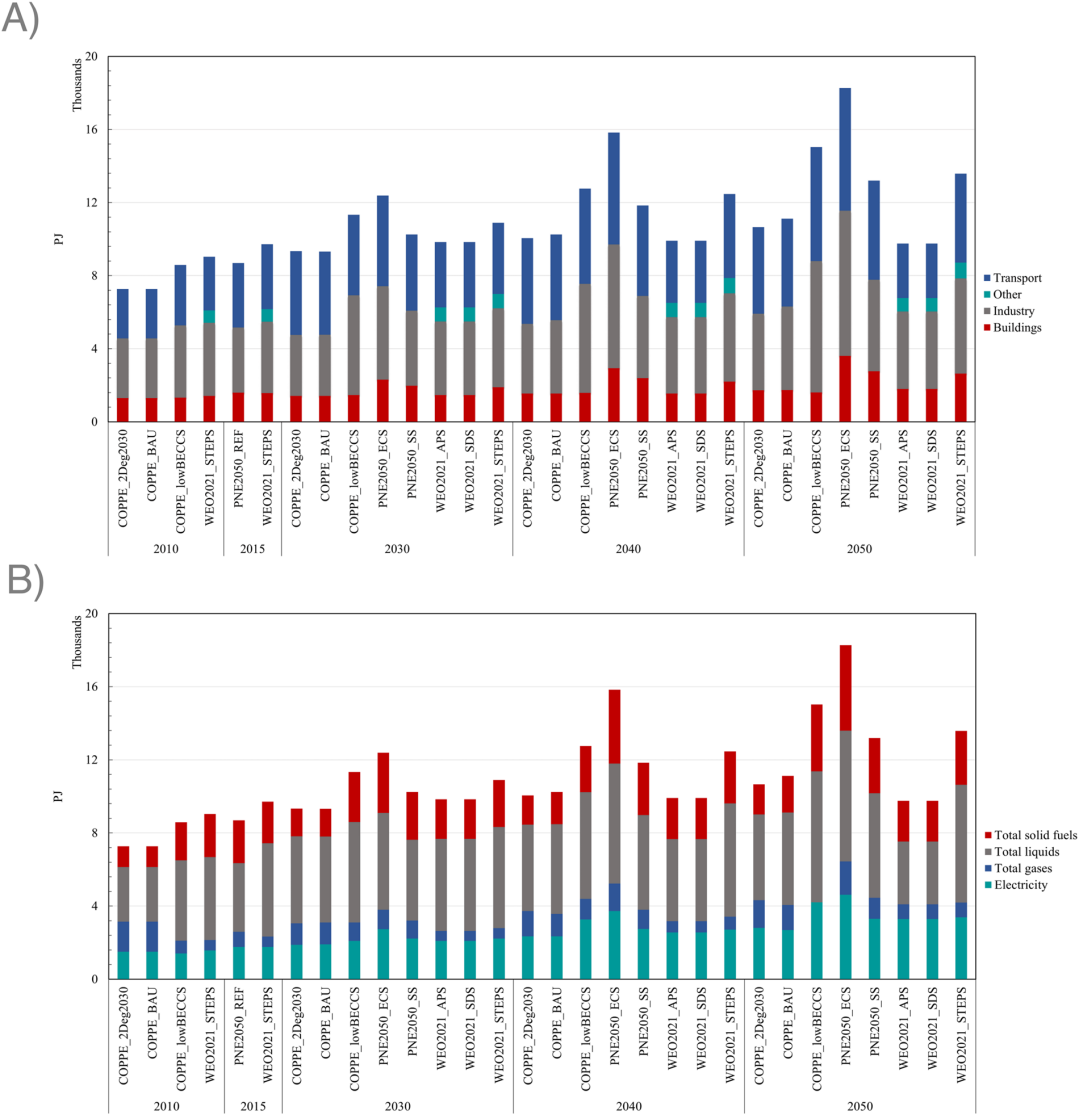
Comparison between demand scenarios by different studies – (**A**) by sector, (**B**) by carrier.

At the top of Fig. 6 presents the total final energy consumption by the combined sector for “Transportation”, “Industry”, “Buildings”, and “Others”. The COPPE and EPE scenarios do not report the consumption of “Others”. EPE’s PNE2050 indicates that the three reported sectors account for more than 80% of final energy consumption and will continue to be just as important in the long term. “Others” basically considers energy consumption associated with agriculture and livestock. The average final energy consumption in 2015 for the three most important sectors was 7.9 PJ, increasing to 10 PJ in 2030, and reaching 12.4 PJ in 2050. In the long term, there are important variations depending on the scenario, as detailed in Supplementary Tables S4–S6.

At the bottom of Fig. 6 shows the total final energy consumption by integrated energy type for each scenario considered. In the long term, electrification is increasingly critical in the three sectors with the highest consumption. Electricity consumption represented 19% in 2010, and the average between the scenarios indicates that it could reach 21% in 2030 and 28% in 2050. There are essential differences in the role that electrification could play between scenarios, especially in the transport sector, where electrification may decline depending on the advancement that bioenergy with carbon capture and storage (BECCS) technologies may have in the long term. With a significant development of BECCS, total electricity consumption would be approximately 1.8 PJ in 2050, while with a conservative development of BECCS, total electricity would be approximately 4.4 PJ. For more details on the consumption of other solid, liquid, and gas energy, please refer to Tables S4–S6.

### Inflow of hydropower plants

Hydropower is an essential sustainable energy source, particularly in developing countries such as Ecuador, China, and Brazil. It constitutes the largest share of renewable energy sources and the total generation matrix. With the increasing penetration of vRES in the power system, properly representing hydropower in the power system analysis becomes crucial. This is because run-of-river (mostly low-head) hydropower plants usually provide (in contrast to vRES) constant base load, and other hydropower plants with reservoirs, or even pump-storage units can be used for equalising the volatile load by vRES^35^. The theoretical output of electric power from hydropower plants is determined by the combination of available water flow and available head height at each location^36^. The power output is usually limited to the plant’s nameplate capacity at the turbine’s maximum flow rate.

ONS regulates the capacity of the reservoir system and dispatches 163 plant units of different types, including ten reservoirs, 92 run-of-river units, 60 hydropower plant units with reservoirs, and one pumped storage^37^. The unit here is a cluster of hydropower plants dispatched by ONS. The hydraulic operation of the reservoir systems in Brazil can provide about 210 TWh storage energy (expressed as MWmês in the original dataset, where 1 MWmês = 720 MWh/month), of which about 69% is located in the southeast/central of the SIN, followed by the northeast region at about 18%. The southern and northern regions of the SIN account for 7% and 6% respectively^38^.

#### Data collection

Frequently, the energy system models account for known inflows and outflows to model hydropower^39^. Furthermore, hydropower is typically represented in the energy system optimisation model with its historical operating patterns (time series) that indicate restrictions on the hydropower system in the year from which the data was gathered^40^.

ONS publishes daily, weekly, and monthly resolved time series separately about the inflow of the reservoirs, categorized as Affluent Natural Energy (Portuguese: Energia Natural Afluente, ENA) and Stored Energy (Portuguese: Energia Armazenada, EAR) separately^41^. These datasets are available at different levels of aggregation, such as by reservoir, subsystem, basin, or equivalent energy reservoir (Portuguese: Reservatório Equivalente de Energia, REE). These data are continuously updated.

ENA refers to the energy flowing to the hydropower system at aggregated levels. The EAR is a value that reflects the reservoir levels and how much energy they can still produce. The ENA and ERA datasets have been used in several studies, such as^42^ and^43^. The absence of metadata makes it unclear which attributes from the original dataset are utilised.

The ENA dataset has two attributes: the gross ENA and the storable ENA. Gross ENA is the energy generated by the power plant system operating at an assumed 65% of the useful operating level (i.e., the natural water flow into the reservoir). On the other hand, storable ENA is equal to the difference between the natural inflow and the flow into the reservoir. The quantity of EAR represents the energy associated with the amount of water stored in the reservoir, which can be converted into power generation for the plant itself and all the plants downstream of the cascade. The maximum ERA represents the storage capacity of the system at full load. In comparison, the downstream subsystem considers using water from the reservoir to generate energy at the downstream power plants in different subsystems. Since ENA reflects the potential power generation of the hydroelectric power, which is calculated by the volume stored in the reservoirs at their respective operating level, we use this attribute to be the inflow to the hydropower system.

The ONS makes ENA data available at multiple aggregated geographic levels. We need access to the hydro station cascades at each hydropower plant’s resolution level to use the ENA data at the federal-state level. However, information about the interrelationship between individual hydropower plants and the aggregated level of reservoirs, basins or REE is not available. Even though ONS discloses the basins where the power stations are situated, ONS only provides the name of the hydropower plants. Because of the inconsistent nomenclaturebetween datasets, our effort to match strings to determine the precise hydropower station in the prior dataset was unsuccessful. As a result, we use the dataset of ENA spatially resolved by electric region to represent the hourly feed-in to the hydropower plants, aggregated at the federal-state level.

#### Data processing

The ENA data used is daily resolved and is given in a unit MW_month (Portuguese: MWmês). This data is converted to MWh as it is equivalent to the 720 MWh/month^42^.

**Fig. 7:**
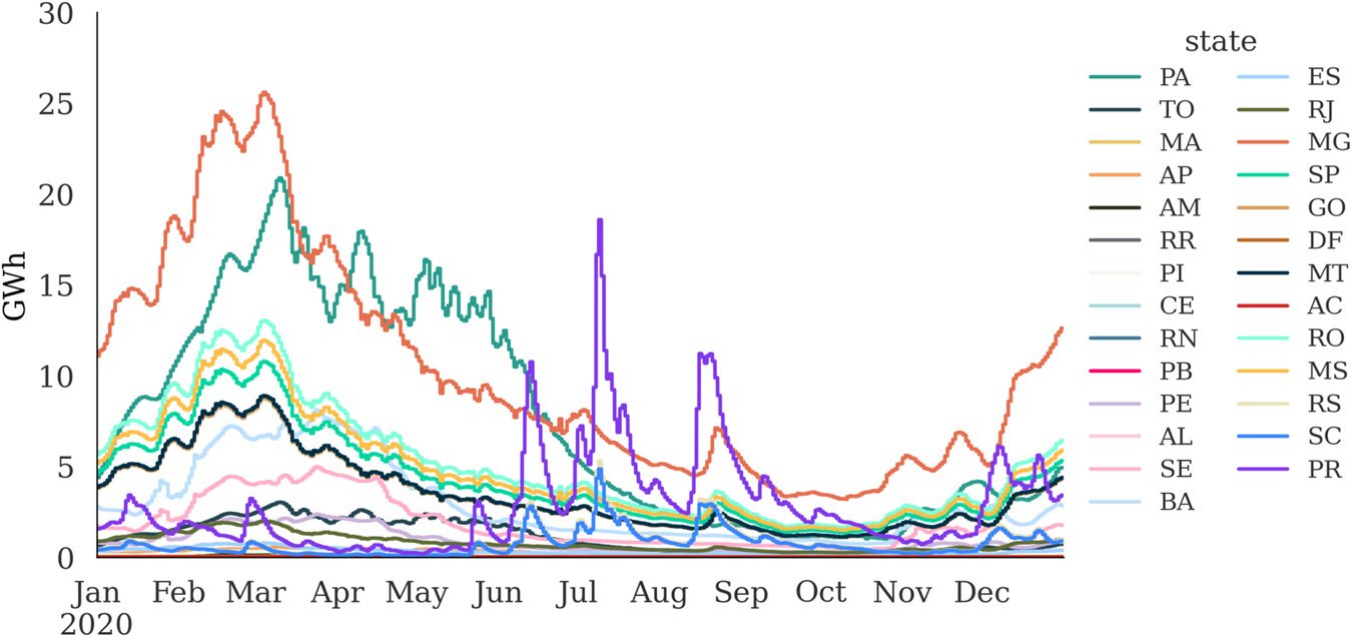
Per federal state inflow for the reference year 2020. Note: allocation is based on the installed capacity (phase is operation) of hydropower plants in the reference year.

To represent the inflow of the hydropower plants in the federal state, we assume that the inflow of the hydropower plants in each federal state correlates to the installed capacity of the reference year. The installed capacity is obtained from the Subsection of Power plants including the hydropower plants of different sizes. As a result, we can visualise the inflow of the hydropower plants at the aggregated level in Fig. 7. The installed capacity for a given reference year has two operating states–operating and planning. Hence, the inflow dataset provided in this study can be allocated either by installed capacity or by the total value of installed and planned capacity.

Finally, we distribute the daily inflows equally to each hour of the day to obtain the hourly inflows.

### Variable renewable potentials (wind and solar)

For planning future energy systems, knowledge of the technical generation potential of vRES is essential. In particular, this includes geo-referenced data on the nominal installation capacity that can be installed in a specific area, along with an hourly generation time series due to the intermittent generation.

#### Data collection

The global resource assessment tool, Energy Data Analysis Tool (EnDAT), assesses the renewable energy generation potential of different technologies such as PV, onshore, and offshore wind turbines. The methodology is developed for Europe as described in^44^ and adapted for global application in^45^. So far, EnDAT is only available internally at the German Airspace Center (DLR). However, it is currently being revised, translated into Python, and prepared for open-source publication, slated for the first quarter of 2023. EnDAT requires inputs of weather resource maps at an hourly temporal resolution and a spatial resolution of 0.09° × 0.09°, along with static land cover maps at a resolution of 0.09° × 0.09°. As output, EnDAT provides (1) spatially resolved maximum generation capacity and (2) relative profiles of hourly power feed-in from wind and solar energy. The spatial output resolution of 0.09° × 0.09° is aggregated to the level of administrative regions, namely, the federal-state level in this paper.

For calculating the installable capacity, two sets of maps are used. One serves as areas of exclusion (cf. Table 11), while the other serves as suitability criteria to determine the share of the remaining available area (cf. Table 12). The spatial land cover maps are based on the Copernicus land cover dataset^46^, the global lakes and wetland database^47^, IUCN protected area categories^48^, and a digital soilmap of the World (for dunes, glaciers, saltpans)^49^. The roughness length is calculated using the land cover maps and a roughness lookup table^50^. Furthermore, we use the spatio-temporal resolved maps from the ERA-5 dataset^51^ to generate the feed-in time series. It contains hourly resolved data for Global Horizontal Irradiance (GHI), wind speed, and temperature at a 31 km spatial resolution.

**Table 11:** Utilizable areas for the EnDAT analysis.

Criteria	Map	PV	Wind onshore	Wind offshore
inclusion	slope (°)	*m* < 45°	*m* < 45°	—
inclusion	distance to settlement (km)	1 < *m* < 1000	1 < *m* < 1000	—
inclusion	elevation (m)	0 < *m* < 5000	*m* < 5000	−50 < *m* < 0
inclusion	average wind speed (m/s)	—	0–50	0–50
inclusion	distance to coast (km)	—	—	5 < *m* < 115
inclusion	mining (0..1)	*m* = 0	*m* = 0	—
inclusion	salt/sand/ice (0..1)	*m* = 0	*m* = 0	—
exclusion	protected areas	*m*∈{1,…, 6}	*m*∈{1,…, 6}	*m*∈{1,…, 6}
exclusion	wetland	*m*∈{1,…, 10}	*m*∈{1,…, 10}	—

*m* denotes the value constrained according to the map, while the provided integer categories are excluded.

**Table 12:** Suitability factors for the EnDAT analysis.

Map	PV	Wind onshore	Wind offshore
bare	0.6	0.3	—
crops	0.24	0.15	—
grass	0.6	0.15	—
moss	0.6	0.3	—
shrub	0.6	0.15	—
forest	—	0.05	—
urban	0.024	—	—
marine water body	—	—	0.4

The land cover maps are given in shares from 0 to 1 and are not mutually exclusive. Map data is taken from the Copernicus dataset^46^.

#### Data processing

We use geometric constraints to calculate the maximum installation density, i.e., taking into account the wake for wind and the maximum shading of the assumed module angle for PV during the winter solstice. The density is restricted by the available area, considering information on the land cover of the area, such as bare ground, crops, grasslands, mosses, shrubs, forests, urban area, and roughness, as well as excluded areas, such as distance from settlements, elevation, mining sites, protected areas, glaciers, slopes, wetlands, and water depth for offshore winds. By fulfilling any exclusion criteria or violating one of the inclusion criteria, we create exclusion masks to restrict the calculation to the desired areas in the potential analysis. The resulting exclusion criteria are provided in Table 11.

Next, suitability factors (cf. Table 12) are used to obtain the share of area available per land-cover type that can be used to install a particular technology. Therefore, for each power generation technology, a projection of the techno-economical parameters into the year 2050 is performed (cf. Table 13). The potential for PV capacities is determined for rooftops, facades, and other surfaces in urban and open areas where ground-mounted PV is installed. At the given resolution, one pixel can have more than one land cover type. Hence, the shares of each pixel are considered additive. The resulting installable capacity is an averaged value.

**Table 13:** Technical parameters for the different generation technologies in EnDAT.

Category	Parameter	Unit	Value
PV	power reduction	1/K	−0.005
PV	*η*_*module*_	—	0.26
PV	*η*_*rest*_	—	0.91
PV	availability	—	0.98
all wind onshore	nacelle height	m	112
all wind onshore	rotor diameter	m	165
all wind onshore	distance factor	—	6
all wind onshore	wind shading loss	—	0.85
all wind onshore	availability factor	—	0.982
wind onshore weak	nameplate capacity	kW	3630
wind onshore medium	nameplate capacity	kW	5330
wind onshore strong	nameplate capacity	kW	10550
wind offshore	nacelle height	m	150
wind offshore	rotor diameter	m	200
wind offshore	distance factor	—	6
wind offshore	wind shading loss	—	0.85
wind offshore	availability factor	—	0.95
wind offshore	nameplate capacity	kW	10000

The subsequent evaluation of the feed-in time series is performed based on assessing the maximum generation capacity. Weather data are converted into power generation in each pixel and weighted by the spatial distribution of the installable generation capacities. For PV, feed-in time series are computed based on the module angle, orientation, and the hourly sun position at a temporally resolved GHI, Direct Normal Irradiance (DNI), and temperature profile. ERA5 provides only GHI, so we use the python library. pvlib^52^, to derive the DNI from the global irradiance data. The wind feed-in time series considers the hourly wind speed (corrected at hub height using the local roughness) and power curves of turbines^44,45^. Finally, generation capacities and time series are spatially aggregated to a defined region–Brazil’s federal-state level.

**Fig. 8:**
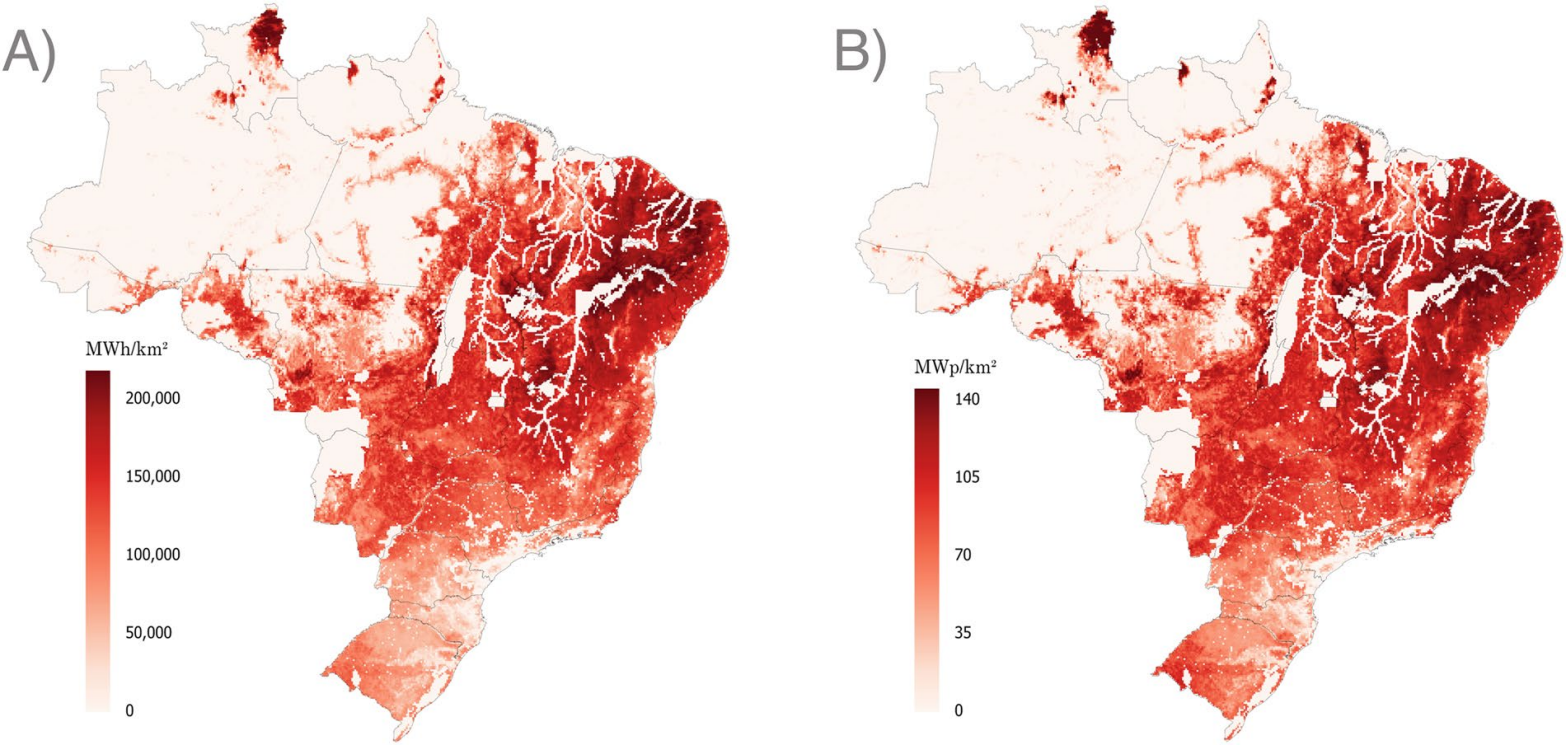
Maps of PV power generation potential for the reference year 2019 – (**A**) Annual generation, (**B**) Installable capacities. Each map combines the potential for urban and open field installation and generation.

**Fig. 9:**
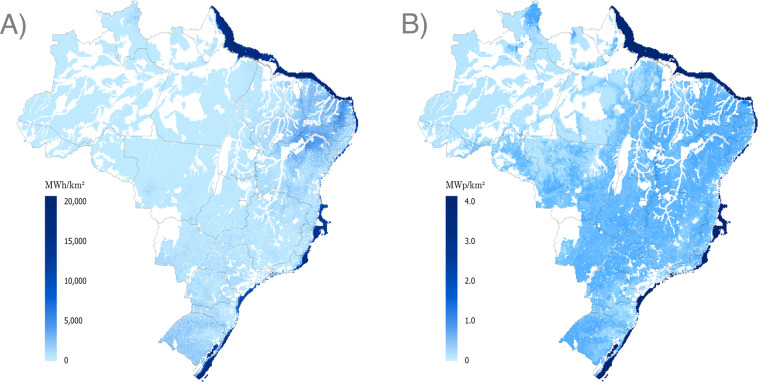
Maps of wind generation potential for the reference year 2019 – (**A**) Annual generation, (**B**) Installable capacities. Onshore wind and offshore wind are combined in each map.

The map of installable capacity (in MW/km^2^) and the annual power production map (in MWh/km^2^) illustrate the resource maps obtained from the Brazilian potential analysis. Figure 8 indicates PV generation and Fig. 9 illustrates wind generation, where geographical features such as bodies of water or rain forests are visible.

### Cross-border electricity exchanges

In addition to the national electricity transmission, SIN connects Brazil to Uruguay, Argentina, and Venezuela for importing and exporting electricity to these countries. Annual power imports remain modest, accounting for only 0.04% (0.60 TWh) of total annual energy consumption, with most of the imports happening between May and November.

#### Data collection

ONS publishes hourly historical cross-border flows with Uruguay and Argentina^53^, with the time series data available for the period 1999–2020 for Argentina-Brazil and 2000-2020 for Uruguay-Brazil. We acquired the dataset in July 2021.

#### Data processing

The cross-border power exchange data from ONS have gaps in the time series. In particular, the data for Uruguay-Brazil has missing values for each year except 2018–2020. Most of the data is missing for 2000–2003, and 2.5% of values are missing in 2016 and 0.3% in 2014. The Argentina-Brazil dataset has one or two missing values in each year except 2019–2020. For 2008, 2009, and 2016 we observe missing shares of missing values of 6.6%, 12.1%, and 2.5%, respectively. To be consistent with the time frame of other datasets, only the time series for 2012–2020 are selected further processing. Missing values are mainly filled with the value of the same point in time from the previous week, with the previous hour being used for the rest.

**Fig. 10:**
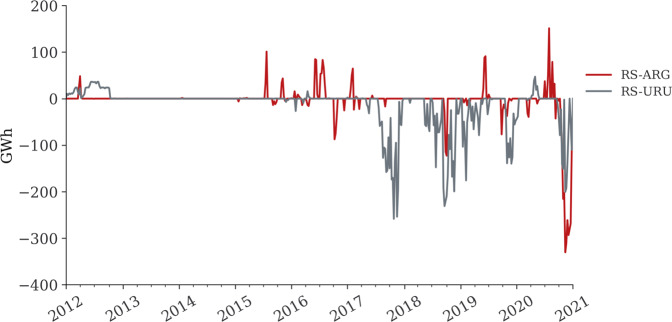
Weekly cross-border electricity transmission from Brazil-Uruguay (RS-URU) and Brazil-Argentina (RS-ARG). Note: The dataset provided is hourly.

The substations for both transmission lines are located in the Rio Grande do Sul (RS) in the Brazilian territory^54^. We manually label the IDs for federal states using two characters, whereas, for foreign substations, we use three characters (URU for Uruguay, ARG for Argentina). Accordingly, the transmission are labelled as RS-URU and RS-ARG, as illustrated in Fig. 10.

## Data Records

**Fig. 11:**
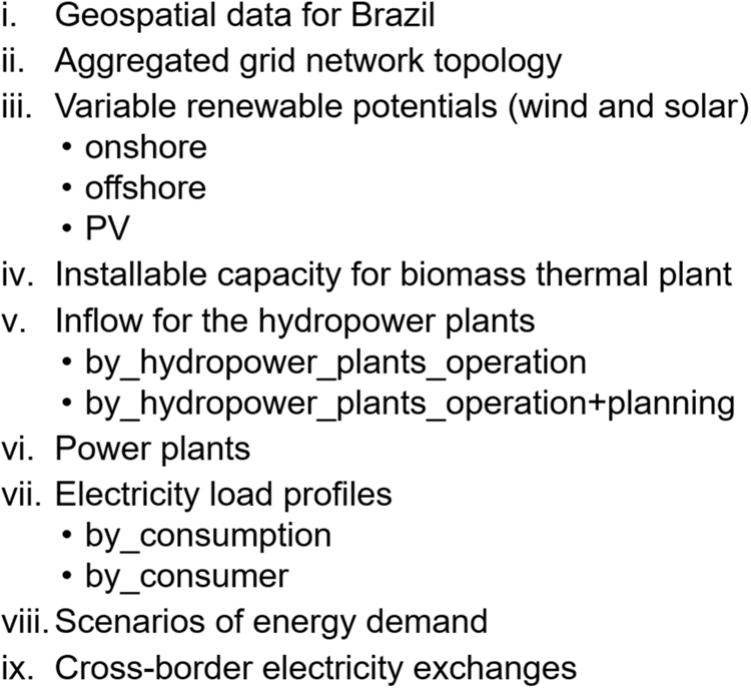
Folder structure of data records on Zenodo^55^.

The dataset provided in this paper is publicly available for download from the repository^55^. The download file contains nine directories, each representing a subset. Figure 11 illustrates the folder structure. The data files within each directory are in a standard format of CSV, except for geospatial data for Brazil. All data are spatially resolved at the ISO 3166-2 level and temporally resolved in hours. The time series files are provided for the reference years from 2012 to 2020.

### Geospatial data for Brazil

This folder contains a shapefile, which can be opened in geographic information system software. The CRS is EPSG:4087. The description of the entities is detailed in Table 14. This data determines the nodes used for the entire dataset provided in this paper, i.e., the abbreviations of the federal states.

**Table 14:** Metadata of the records for (i) Geospatial data for Brazil.

*node_epsg4087.shp*		
*filed*	*type*	*description*
name	string	Abbreviation of federal state
state_full	string	Full name of the federal state in Portuguese
x	number	The latitude of the polygon centre geometry of the federal state, and CRS is EPSG:4087
y	number	The longitude of the polygon centre geometry of the federal state, and CRS is EPSG:4087

### Grid network topology

We provide two files–one including only the topology of the operational network (file name: EPEWebmap_equivalent_grid_aggregate_by_state_only_operation.csv), and the other additionally covering the planned network (EPEWebmap_equivalent_grid_aggregate_by_state_operation_and_ planed.csv). Table 15 explains the attributes. The voltage is not shown here because it is an equivalent network for which the net transfer capacity is calculated.

**Table 15:** Metadata of the records for (ii) Grid network topology.

*EPEWebmap_equivalent_grid_aggregate_by_state.csv*		
*filed*	*type*	*description*
node0	string	start node
node1	string	end node
transfer capacity	number	transfer capacity between the start and end nodes, in MW
efficiency	number	transmission efficiency between the start and end nodes, assuming an efficiency of 1 for HVDC lines
name	string	The data processing produces a string that helps to trace each transmission line in the original dataset (EPE Webmap) by line name. The different line names are connected by the character “_”.
length	number	length of the representative transmission between the start and end nodes
carrier	string	the type of the line, either AC or HVDC

### Variable renewable potentials (wind and solar)

The data are organized in a directory structure containing CSV files. Three generation technologies (wind onshore, wind offshore, PV) are in three directories: onshore, offshore, and solar_pv.

These directories contain the installable potentials and the yearly time series. The installable potentials are named as EnDAT_ < TECH_NAME > _installable_capacity.csv and the time series as EnDAT_ < TECH_NAME > _per_unit_generation_weather_year_ < YYYY > .csv. The text “YYYY” corresponds to the weather year. The installable capacities contain the region abbreviation and the installable capacity in MW. The time zone of the time series is UTC + 0.

The first column of the generation time series data represents the hourly timestamp in the format of YYYY-MM-DD HH:00:00. The subsequent columns are the unit generation for each federal state, and the column names are the abbreviations of the respective federal states.

### Installable capacity for biomass thermal plants

The subset includes files of the installable capacity records, one per year, named biomass_geographic_potential_reference_year_ < YYYY > .csv. The text “YYYY” corresponds to the reference year. Table 16 reports the details of the information provided by each record.

**Table 16:** Metadata of the records for (iv) Installable capacity for biomass thermal plant.

*biomass_geographic_potential_reference_year_YYYY.csv*		
*filed*	*type*	*description*
state	string	abbreviation of federal states
value	number	the installable capacity, in MW
reference_year	number	reference year, i.e., YYYY
type	string	power plant type–“biomass”
phase	string	Operational status. All values here are “potential”, indicating the installable capacity, which is used to differentiate the status in the data of power plants.

### Inflow for hydropower plants

The inflows to the hydropower plants in each federal state are obtained separately from two allocation parameters related to the operating status of the total installed capacity. Therefore, there are two subdirectories under this folder, namely, by_hydropower_plants_operation + planning and by_hydropower_plants_operation. Each subdirectory includes nine files for each reference year. Each file lists the federal state in its columns, with each row representing the hourly inflow, measured in MWh, for that federal state throughout the year at the timestamp YYYY-MM-DD HH:00:00. The time zone of the time series is UTC-3 (Brasília Time).

### Power plants

Table 17 presents the description of the data attributes. The information on the installed capacity of power plants in each federal state is recorded in relation to the reference year, with each file representing a record for a specific reference year.

**Table 17:** Metadata of the records for (v) Power plants.

ANEEL_powerplants_per_state_per_type_reference_year_YYYY.csv		
filed	type	description
state	string	abbreviation of federal states
type	string	the type of power plants type–biomass, solar_pv, on_wind, mini_hydro, small_hydro, hydro, nuclear, coal, gas, oil
phase	string	operation status–operation or planning
value	number	capacity in MW
reference_year	number	reference year, i.e., YYYY

### Electricity load profiles

It includes two subdirectories, by_consumer and by_consumption. This is related to the disaggregation of the original dataset, as presented in the Subsection of Electricity load profiles. Under each subdirectory are hourly load curves for each reference year, as Table 18 details.

**Table 18:** Metadata of the records for (vii) Electricity load profiles.

*Hourly_electricity_demand_per_state_YYYY.csv*		
*filed*	*type*	*description*
time	string	the time stamp, DD.MM.YYYY HH:00:00, time zone is UTC-3 (Brasília Time).
state	string	abbreviation of federal state
value	string	load value in MW. Note: As this is derived from the grid operator ONS, it includes the physical loss of SIN.

### Scenarios of energy demand

We provide energy demand data (XLSX format) aggregated by energy carrier and end-use sector for PNE2050 and COPPE as the attributes of the records are detailed in Table 19. Due to legal issues, we can only show the IEA data in Fig. 6. To speed up the data processing, we provide the data in CSV format encoded in UTF-8.

**Table 19:** Metadata of the records for (viii) Scenarios of energy demand.

*Energy_demand_scenarios_by_sector_by_energy_carrier.xlsx*		
*filed*^*i*^	*type*	*description*
Publication	string	the source of the data
Scenario	string	the full name of the scenario
Region	string	the name of the country
Category	string	the indication of the data category. As it is the dataset of energy demand, it is “Energy”.
Product	string	the energy carriers with aggregation. Values are “Total”, “Electricity”, “Total liquids”, “Total gases”, and “Total solid fuels”. Note: “Total” is the sum of the remaining energy carriers.
Flow	string	end-use sectors with aggregation. Values are “Total final consumption”, “Transport”, “Buildings”, “Industry”, and “Other”. Note: “Total final consumption” is the sum of the remaining end-use sectors.
Unit	string	unit of the demand value, i.e., PJ.
Year	numeric	year. Values are “2010”, “2015”, “2030”, “2040”, and “2050”.
Value	numeric	value of the demand. The decimal point is written in ‘,’.
Alias	string	The alias of the scenario used for plotting. It has the format XXXX_YYYY. XXXX is the abbreviation of the study, i.e., “WEO2021”, “PNE2050”, “COPPE”. YYYY indicates the abbreviation of the scenario name, Table S4–S6.

### Cross-border electricity exchanges

Under this folder, there is a single file named international_transmission _RS-URU_RS-ARG_2012-2020_hourly.csv. It stores records of cross-border electricity imports and exports between Brazil and its neighbours for the 2012–2020 timeframe. A description of the records on the file is presented in Table 20.

**Table 20:** Metadata of the records for (ix) Cross-border electricity exchanges.

*Cross-border_transmission_RS-URU_RS-ARG_2012–2020_hourly.csv*		
*filed*	*type*	*description*
time	string	the hourly time stamp, YYYY-MM-DD HH:00:00, time zone is UTC-3 (Brasília Time).
node0	string	start node with Brazilian federal state abbreviation, namely, RS
node1	string	end nodes for neighbouring country abbreviations, i.e., ARG and URU
power	number	electricity exchanged, MW

## Technical Validation

Most of the original datasets are taken directly from the official Brazilian database. For this reason, the datasets provided in this paper have not undergone additional validation. However, it is necessary to note that spatially aggregating Brazil’s power transmission network to a network model of interconnected federal states implies deviations in the resulting power flows. For validation, reference data from power-flow analyses of the fully-resolved network is required, ideally for a multitude of grid uses. Since these use cases strongly depend on the power plant dispatch and future load patterns, a validation would call for a power system model for the fully-resolved network. However, setting up such a model for validating has been beyond our capabilities.

Another exception requiring validation is the dataset described in the Subsection of Variable renewable potentials. The technical validation is approached with two data sources: (1) observations of site-specific power generation from a set of real-world PV plants and wind farms in 2018^56^, and (2) country-wide power generation indicators from global databases for 2019, namely the Global Wind Atlas (GWA)^57^ and the Global Solar Atlas (GSA)^58^.

### Solar feed-in

The spatial distribution of PV plants is shown in Fig. 4. We gather the installed capacity for each PV park based on^15,59^. Of the 17 PV parks, we use 12 for further analysis. The Pearson correlation is calculated to determine whether the temporal profiles generated by simulation and observation are similar. Table 21 presents the average correlation between the simulated data and the reference for each PV park, which is approximately 0.8. The deviation can be explained by the fact that the orientation and inclination of the reference PV installation cannot be determined from available data sources, such as aerial images. As the effect of the orientation under small module inclinations is minimal, this aspect is not considered in our assessment with EnDAT. By default, EnDAT calculates an ensemble of solar power plants with a southern orientation facing east and west at 60° away from the south.

**Table 21:** Correlation between PV site power generation and EnDAT simulation results for these sites.

Plant name	corr_Pearson_
Fontes Solar I	0.92
Fontes Solar II	0.92
Assu 5	0.87
Conjunto Fotovoltaico Bom Jesus	0.86
Conjunto Fotovoltaico Ituverava	0.88
Conjunto Fotovoltaico Lapa	0.85
Conjunto Fotovoltaico Pirapora 2	0.83
Conjunto Fotovoltaico Nova Olinda	0.86
Conjunto Fotovoltaico BJL Solar	0.62
Conjunto Fotovoltaico Floresta	0.82
Conjunto Fotovoltaico Horizonte MP	0.76
Conjunto Fotovoltaico Guaimbe	0.78

The quantity for country-wide validation with the GSA^58^ is performed by converting solar resources, namely DNI and GHI, into power generated per unit of capacity of pre-defined PV power plants over the long term, called PVOUT. The solar resources are obtained by Solargis are compared to those from ERA5 reanalysis data used by EnDAT. Since the GSA provides raster data with 1 km resolution, we upscale it to match the 0.09° resolution of EnDAT using the nearest neighbour method. We use the mean bias error (MBE) to evaluate the difference in levels of overestimation or underestimation. The comparison of PVOUT derived from EnDAT and the GSA shows an MBE of −7% and −36% PV in urban and open areas, respectively. Especially, the deviation increase with the distance to the equator. The result indicates that EnDAT overestimates the PVOUT in comparison with GSA. The deviation can be attributed to the differences in solar resource data^60^ and is considered reasonable.

### Wind feed-in

The spatial distribution of wind power plants is shown in Fig. 4 (only onshore wind).

As a validation dataset, we use the observed hourly electricity production in 2018 published by ONS^56^ of several wind farms. The installed capacity, hub height, location, and turbine type of each wind farm are gathered from references^15,61^. Of the obtained eleven Brazilian wind farms, we use seven for further analysis due to data inconsistencies. As detailed in Table 22, the correlation between real-world wind farms and EnDAT-simulated generation time series ranges from 0.23 to 0.58. The deviation may be due to the fact that  the potential analysis approach of EnDAT does not account for local wind effects caused by elevation, which could result in gaps in correlation. However, most existing wind farms are located in areas highly influenced by local wind phenomena. Several of the investigated wind parks are located on the coastline where hot winds can cause temperature differences between land and sea, superposed with nearby elevation changes inland of the wind parks. Other sites are located on plateaus in hilly terrain. The weather data used by EnDAT–wind speed–originates from ERA5 reanalysis data, which has a resolution of 31 km at the equator, and is represented as the grid average. On small geographical and temporal dimensions, however, observations of wind speed can differ due to the local terrain, vegetation, and built environments^51^. The wind speed data from ERA5 may not accurately describe wind speed in highlands or valleys.

**Table 22:** Correlation of wind power generation between wind farm observations and EnDAT simulations.

Plant name	corr_Pearson_
Praia Formosa	0.44
Icaraizinho	0.51
Malhadinha 1	0.23
Alegria II	0.32
Alegria I	0.38
Elebras Cidreira 1	0.58
Xangri-LA	0.52

The data of GWA 3.0^57^ is derived from the same reanalysis data, ERA5 as we do. However, the GWA only provides average wind speed, and average power density at five different heights (10m, 50m, 100m, 150m, and 200 m) and average capacity factors (CFs) for three turbine classes as defined by the International Electromechanical Commission (IEC). To compare the CFs for IEC class I and III from GWA with EnDAT-simulated results, we upscale the GWA data (spatial resolution of 250 m) to EnDAT’s spatial resolution of 0.09° for Brazil using the nearest neighbour method. Compared to GWA, our CFs for onshore wind are lower and for offshore wind are higher, although we align our technical specifications with the GWA’s assumptions for this validation–Vestas V112 turbines for IEC class I and V136 turbines for IEC class III. In particular, the MBE between CFs calculated for onshore wind between EnDAT and the GWA is 17% for IEC Class I and 18% for IEC Class III. The MBE for offshore wind is 17% in IEC Class I and 14% for IEC Class III. However, we are unable to identify all the factors contributing to the differences between our data processed and the GWA due to barriers in accessing details on assumptions made for the GWA.

### Conclusion

To summarise, our simulations correlate better with real-world PV generation than onshore wind generation at a spatial resolution of 0.09°. EnDAT calculates a higher PV generation compared to the GSA. The onshore wind power potential obtained by EnDAT is lower than the GWA, while the offshore wind power potential calculated by EnDAT is higher It is essential to highlight that the data we provide for PV and wind power is aggregated to large geographical areas, i. e. at the federal-state level. For this geographical dimension, appropriate validation data still need to be included, as available validation data is limited and often site-specific. Our data shows better agreement with simulated data from GSA and GWA, which rely on much higher resolved resources data but only provide CFs instead of time series of power generation. However, downscaling may be necessary when using the regionalised results from EnDAT.

## Usage Notes

The dataset provided in this paper consists of multiple CSV files, and can be loaded using software capable of handling such files. The use of is self-explanatory, which can serve as input to any energy system model. With its high-resolution (hourly and for the 27 federal states of Brazil), the data enables the emulatiion of the Brazilian power system the represention of Brazil in a global energy system model at a sufficient resolution.

However, it is not appropriate to compare historical annual trends in data where the reference year is determined by the installed capacity. This applies to data, for instance, (iv) Installable capacity for biomass thermal plant and (v) Inflow for the hydropower plants. This is because most of the date information in the original dataset is missing, as described in the Subsection of Power plants.

To achieve the objective of providing a reliable and open database for modelling the Brazilian power sector, we make available the evolution of electricity consumption by sector until 2050 in Subsection of Scenarios of energy demand. It is necessary to learn the principal premises of each scenario to understand the dynamics of the evolution of electricity consumption, presented in Supplementary Tables S4–S6. For example, the dispute between electrification and biofuels (aggregated to total liquids) in the transport sector. Therefore, to better comprehend the role of electrification in each sector and the intersectoral dynamics, the evolution of the consumption of additional energy carriers in each sector until 2050 is also presented in complementary form (cf. Figure 6). It should be noted that the PNE2050 data may contain numerical deviations arising from the extraction of number from the charts.

In this paper, we highlight data with harmonized resolution. Although the available data for 27 federal units contribute to the spatial resolution of the Brazilian energy system model compared to the data currently used, the intent of harmonization may limit the study of energy systems at a higher resolution. Therefore, we leave the code open, which is documented to the best of the author’s knowledge. Under the “resources” folder, users can find the processed data before aggregation to 27 nodes. For example, the data for power plants in under the project folder power_plantsresourceconvert_ANEEL_geolocation_added _state_updated_2021_06.csv.

## Supplementary information

Supplementary Information

## Data Availability

Direct use of our provided datasets is available on Zenodo^55^ The source code used for data collection, processing and analysis is also on Gitlab^17^. The data processing is performed using Python 3.9 and the necessary toolboxes, such as Pandas and Geopandas. The data collection process is fully described in the paper. By open-sourcing the code, we aimi to provide the most relevant information for integrating the dataset into energy system models. Although step-by-step tutorials could also be helpful for this purpose. However, we think such information is best conveyed through the source codes^17^. We regret that we cannot provide scripts for the vRES potential data. The data of vRES potential is created by the EnDAT framework, which is in the process of being open-sourced and only available within DLR. For those data for which the license is “citation”, we have been permitted to redistribute the data after modifying it for this paper. We do not, however, have permission to publish their original data. We will continue to update this dataset and apply this dataset to further energy system studies. We encourage readers to contribute to fill in the gaps and improve the hypotheses of this dataset mentioned in the paper.
