# Supplementary material for: Harmonized and Open Energy Dataset for Modeling a Highly Renewable Brazilian Power System
Source: Sci Data. 2023 Feb 22;10:103. doi: 10.1038/s41597-023-01992-9 (PMC9946950; doi:10.1038/s41597-023-01992-9)
Supplement: Supplementary file 1 — Supplementary Information [file 41597_2023_1992_MOESM1_ESM.pdf]

Supplementary Material

Subsection	Raw Dataset	Data License
Geospatial data for Brazil	IBGE – Municipal Mesh Data[S1]	ODbL[i]
Grid network topology	EPE Webmap[S2]	ODbL
Renewable potentials (wind and solar)	[S3–S8][ii]	citations[iii]
Installable capacity for biomass thermal plant	[S11]	citations
Inflow for the hydropower plants	ONS – Historical Natural Energy Inflow for Each Region[S12]	ODbL
Power plants	ANEEL-SIGA[S13]	ODbL
Electricity load profiles	ONS – Historical Regional Load Curve[S14], EPE – Statistical Yearbook of Electricity[S15]	ODbL
Scenarios of electricity demand	[S16–S20]	citations
Cross-border electricity exchanges	ONS – Historical Energy Exchanges[S21]	ODbL

Table S1. Summary of original open accessible datasets used in this paper and their license

[i]Open Data Commons Open Database License (ODbL): https://opendatacommons.org/licenses/odbl/1-0/. Law NO. 8777 of May 11th, 2016, establishes the Open Data Policy to guide the federal executive branch in releasing open government data. The main parts of the policy are the Open Data Plan (PDA, in Portuguese, Plano de Dados Abertos) by each federal agency and a Brazilian Open Data Portal. These datasets have an Open Data Commons Open Database License (ODbL), for example, "Geometric mesh of Brazilian municipalities".

- See: "CAPÍTULO I, Art. 3º -IV permissão irrestrita de reuso das bases de dados publicadas em formato aberto" is "unrestricted permission to reuse databases published in open format".
- See: "CAPÍTULO II, Art. 4º Os dados disponibilizados pelo Poder Executivo federal e as informações de transparência ativa são de livre utilização pelos Poderes Públicos e pela sociedade. (Redação dada pelo Decreto nº 9.903, de 2019)" is "the data provided by the Federal Executive Branch and the information of active transparency are freely used by the Public Authorities and society. (Writing by Decree No. 9,903, 2019)."

[ii]The data is generated by the EnDAT[S9,S10]. EnDAT is under the procedure of being open source. The dataset published in this paper is the first Brazilian dataset produced by EnDAT.

[iii]We have been permitted to republish the data after modifications made for this paper, but this does not mean we can make their original data publicly available.

	Existing	Planing	Total
Number of lines	1589[i]	453	2042
Number of substations	735	165	900
Number of power plants	2904[ii]	274	3178

Table S2. Number of the lines, substations, and power plant units in the original dataset[iii]

[i]The majority of them are at 230 kV and 500 kV, whereas the lowest voltage level is 69 kV. There are three 800 kV lines. 115 lines have a length of less than 1 km and seven lines have a length of more than 2000 km.
[ii]It includes hydropower plants, wind onshore, PV, fossil-thermal, biomass-thermal, and nuclear power plants.
[iii]These are the statistics downloaded by the author during April 2021. The statistics may differ slightly from the version as the data is updated continuously.

WEO2021[i]	PNE2050	COPPE
	end-use sector	
Industry	Industry	Industry
Transport	Transport	Transportation
Buildings	Residential, Service	Residential, Commercial
Other	Agriculture, Non-energy use	Other Sector
	energy carrier	
Total	Total	Total
Electricity	Electricity	Electricity
Total liquids	Oil products, Diesel fuel, Other oil products, Fuel oil, Gasoline C, Hydrated ethanol, Kerosene/aviation gasoline	Liquids[ii]
Total gases	Natural gas, Liquefied petroleum gas (LPG)	Gases[iii]
Total solid fuels	Coal, Wood, Wood and charcoal, Sugarcane, Other	Solids

Table S3. Correspondence between different studies

[i]The end-use sector and energy carriers defined by WEO21 used in this study for aggregation
[ii]Includes ethanol, biodiesel and advanced fuels
[iii]Includes natural gas and LPG

<table>
<tr><th>Study</th><th colspan="4">IEA WEO2021</th></tr>
<tr><td>Scope</td><td colspan="4">i) global and regional outlooks, ii) environmental impacts of energy use on emissions and pollutants, iii) impact of policy actions and technological change, iv) investment requirements for the fuel supply chain to meet projected energy demand, v) prospects of modern energy access</td></tr>
<tr><td rowspan="2">Scenario</td><td>Net Zero Emissions by 2050 Scenario (NZE)[i]</td><td>Announced Pledges Scenario (APS)[ii]</td><td>Stated Policies Scenario (STEPS)[ii]</td><td>Sustainable Development Scenario (SDS)</td></tr>
<tr><td>“well below 1.5 °C”[iii], an achievable pathway for the global energy sector to achieve net CO_2 emissions by 2050, meeting key energy-related UN Sustainable Development Goals (SDGs). Fully dependent on the emission reductions of the energy sector.</td><td>all climate commitments made by governments will be met in full and on time, such as nationally determined contributions (NDCs) and longer-term net-zero goals, objectives, intentions</td><td>sector-by-sector assessment to extrapolate current or developing policies and measures of governments around the world</td><td>“well Below 2°C”[iii] integrated scenario accomplishes the central UN Sustainable Development Goals related to energy, namely: i) universal access to affordable, reliable, sustainable, and modern energy services by 2030 (SDG 7), ii) significant reduction in air pollution (SDG 3.9), and ii) effective action to mitigate climate change (SDG 13) to reach net zero global emissions by 2070.</td></tr>
<tr><td>Narrative</td><td colspan="4">For Brazil, the macroeconomic and demographic assumptions used in all scenarios are different. Energy demand forecasts are based on the average retail price of each fuel used in the end-use, generation, and other conversion sectors. End-use prices are derived from the projected international prices of fossil fuels and subsidy/tax levels. The price of CO_2 is different under different scenarios. However, providing the narrative of each end-use sector in Brazil is oppugning.</td></tr>
<tr><td>Model</td><td colspan="4">A hybrid model – the IEA’s World Energy Model (WEM) of the data-intensive global energy system and the Energy Technology Perspectives (ETP) model of technical and economic parameters of energy technologies. Total final energy demand is the sum of energy consumption in each end-use sector. Energy consumption is assessed with considerable sectoral and end-use detail derived from historical data on the inventories of existing energy infrastructure and socioeconomic variables. For example, the number of vehicles in the transportation sector, the industrial sector’s production capacity, and the building’s floor space. The demand for energy services is modelled specifically for each sector. For example, in the residential sector, the demand is divided into space heating, water heating, cooking, lighting, appliances, and space cooling.</td></tr>
<tr><td>Key drivers</td><td colspan="2">Population:
• Annual growth rate (2010-50): 0.3%
• Population in 2050: 229 million
• Urbanisation (% of population) in 2050: 92%
GDP:
• Average growth rate (2020-50): 2.6%
Remaining fossil fuel sources
• Oil
• Nature gas
• Coal</td><td colspan="2">Fossil fuel prices:
• Nature gas
• Steam coal
Carbon price End-use prices
• Fuel end-use prices
• Electricity end-use prices
• Wholesale electricity price
Derivation of a simplified merit order for thermal power plants
Calculation of average marginal cost in each merit order segment
Estimate of wholesale price based on average marginal cost
Subsidies to fossil fuels</td></tr>
<tr><td>Source</td><td colspan="4">S16, S22</td></tr>
</table>

Table S4. A comparative analysis of the energy demand scenarios in Brazil – WEO21 study

[i] Global analysis only, not regional analysis
[ii] No specific result designed to be achieved
[iii] “Well below 1.5 °C” and “well below 2 °C”: goals announced in Paris Agreement, limiting the average global temperature increase by 2100 to 2 °C above pre-industrial levels without temperature overshoot (50% probability)

**Study**	**EPE PNE2050**	
**Scope**	Brazil's national long-term strategy - National Energy Plan (PNE) 2050 - published by Energy Research Office (EPE), outlines the government's strategic long-term vision in an integrated way.	
**Scenario**	Expansion Challenge scenarios (ECS)	Stagnation scenarios (SS)
	It reflects the strong growth in total energy demand - a national average annual GDP growth rate of 3.0%. On average, the total final energy consumption will increase by 2.2% per year from 2015 to 2050, with faster growth (2.5%) in the first 15 years until 2050, more than twice the 2015 consumption.	It reflects a trajectory that keeps per capita energy consumption around 2015 - with a national average annual GDP growth rate of 1.6%. From 2015 to 2050, total energy consumption will grow by an average of 1.4% per year (over 10% in total).
**Narrative**	Consider a more stable economic, political, institutional, and social environment that allows for the completion of important structural reforms that will have a significant impact on the business environment, investment, and productivity, thereby contributing to GDP growth. Overall, total final energy demand is rising, with the share of petroleum products decreasing and the share of electricity increasing, driven by all sectors, but mainly by the residential sector.	
**Model**	The energy demand forecasting methodology consists of three modules - economics study, demand projection, and demand integration. The assumptions used in the specific models for each sector are derived from a discussion of the main moderators and key uncertainties in industry, agriculture and livestock, buildings, services, transport, and meeting electricity and fuel consumption. Long-term economic scenarios are used as one of its main informational inputs. By elaborating sectoral scenarios for agriculture and livestock, services and industrial activities, as well as for infrastructure and mobility needs, the sector-specific model estimates changes in demand by source and by sector at the national level.	
**Key drivers**	Population:<br>• Annual growth rate: 0.3%<br>• Population in 2050: 226 million<br>• Urbanization in 2015: 86%<br>• Urbanization in 2050: 89%<br>GDP:<br>• Annual GDP growth rate (2016-50): 3.0%<br>• GDP per capita: 2.8%<br>Number of households:<br>• inhabitants/household (2015): 3.2<br>• inhabitants/household (2050): 2.3<br>• households (2015): 33 million<br>• households (2050): 98 million	
**Source**	S17	

Table S5. A comparative analysis of the energy demand scenarios in Brazil – PNE2050

Study	COPPE		
**Scope**	Integrated long-term scenarios for Brazil from 2010 to 2050 or 2100, in five-year time steps, using representative time slices. Analyse the competition between technologies and energy sources to meet in a cost-efficient way the demand for energy services under policy and emission targets (modelling for end-use sectors includes the industrial, energy, transportation, residential and commercial, and agricultural sectors)		
**Scenario**	Business as usual (BAU)	2Deg2030	lowBECCS
	It is based on most likely socioeconomic assumptions of the second marker baseline scenario from the Shared Socioeconomic Pathways (SSP2) throughout the century, without additional climate policies after 2010.	It is a mitigation scenario consistent with increasing global warming up to 2 °C without overshooting above pre-industrial levels by 2100. It builds on submitted NDCs actions (unconditional and conditional) up to 2030, and then it transitions (constrained by a national carbon budget) cost-effectively toward a 2 °C pathway. To simulate the scenario, a national (2011 – 2050 accumulated) budget of 22 Gt of CO_2[i], derived from a global budget of 1000 Gt of CO_2 in the period from 2011 to 2100 is used[ii]. This scenario uses the same socioeconomic assumptions as the BAU scenario.	It is a mitigation scenario consistent with an increase in global warming up to 1.5 °C above pre-industrial levels in 2100. The "lowBECCSS" is an "End-of-century budget" scenario. In the near-term (2020-2030), it builds on immediate action following implemented national policies (NPi) as of 2020. In the long-term, the CO_2 pathway is constrained by cumulative CO_2 emissions over the entire century, allowing high-temperature overshoot and global net-negative CO_2 emissions (NNCE) in the second half of the century. A global budget of 400 Gt of CO_2 between 2018 and 2100 is used. This scenario is conservative regarding the role of bioenergy with carbon capture and storage (BECCS) on a global scale. To only consider the sustainable global BECCS potential, this technology is capped at around 8 $GtCO_2$/yr in 2100. This scenario incorporates the middle-of-the-road socioeconomic conditions throughout the century, based on the SSP2.
**Narrative**	These are scenarios used for scientific purposes, which can inform decision-making in the Brazilian Government and globally. The useful energy demands for each consumption sector, the food demands, and the reforestation and deforestation scenarios used are calculated exogenously. For the short-term modelling, official public data is considered, while international scenarios are used as a reference for the long-term. The three scenarios shown contain the same population growth and socioeconomic development trajectory (SSP2) but are subject to different levels of ambition to achieve global climate goals.		
**Model**	It is calculated during the COMMIT project with the BLUES model[iii].		This scenario is calculated with the COFFEE v1.1 global model during ENGAGE project[iv].
**Key drivers**	Population:<br>• Annual growth rate(2015-50): 0.31%<br>• Population in 2050: 226.3 million<br>GDP:<br>• Annual GDP growth rate (2015-50): 2.87%<br>• GDP in 2050 (billion US$): 5370.1	Population:<br>• Annual growth rate(2015-50): 0.31%<br>• Population in 2050: 226.3 million<br>GDP:<br>• Annual GDP growth rate (2015-50): 2.75%<br>• GDP in 2050 (billion US$): 5257.9	Population:<br>• Annual growth rate(2015-50): 0.40%<br>• Population in 2050: 231.9 million<br>GDP:<br>• Annual GDP growth rate (2015-50): 4.01%<br>• GDP in 2050 (billion US$): 7042.9
**Source**	S18, S19		S20

Table S6. A comparative analysis of the energy demand scenarios in Brazil – COPPE

[i] More precisely, it should be the CO_2 equivalent of greenhouse gas emissions

[ii] This global carbon budget represents a high probability (above 0.66) to keep global warming levels below 2 °C by 2100[S23].

[iii] The Brazilian Land-Use and Energy System model (BLUES) is an application of the MESSAGE platform. It is a mixed-integer linear optimization model, which minimizes the total cost of expanding the energy-land system to meet the expected demand for energy services and food. It combines technical, economic, and environmental variables for more than 8000 technologies with imposed constraints (including reforestation and deforestation scenarios) to obtain an optimal solution for the energy and Agriculture, Forest, and Other Land Use (AFOLU) sectors. The document is https://www.iamcdocumentation.eu/index.php/Reference_card_-_BLUES

[iv] The Computable Framework For Energy and the Environment (COFFEE) is a global and multi-sectoral partial equilibrium model with 18 regions (including Brazil) that runs on the MESSAGE platform. It uses the base year of 2010, with a horizon of 2100 in five-year time steps. The model's objective is to assess the potential synergies and trade-offs between energy systems and environmental and climate policy. The model includes all energy and land-use systems, with a hard link between the two. The macroeconomic inputs into the model come from exogenous macroeconomic drivers providing demand growth over time or from the TEA model, which pulls from the SSP database.

References of supplementary material

S1. Instituto Brasileiro de Geografia e Estatística. Malha Municipal: BR_UF, https://www.ibge.gov.br/geociencias/organizacao-do-territorio/malhas-territoriais/15774-malhas.html?=&t=downloads (2021).

S2. Empresa de Pesquisa Energética (EPE). Sistema de Informações Geográficas do Setor Energético Brasileiro: Linhas de Transmissão, https://gisepeprd2.epe.gov.br/WebMapEPE/ (2020).

S3. Buchhorn, M. *et al.* Copernicus Global Land Service: Land Cover 100m: collection 3 : epoch 2015: Globe (2015). Zenodo https://doi.org/10.5281/zenodo.3939038.

S4. Lehner, B. & Döll, P. Development and validation of a global database of lakes, reservoirs and wetlands. *J. Hydrol.* **296**, 1–22, https://doi.org/10.1016/j.jhydrol.2004.03.028 (2004).

S5. Dudley, N. *Guidelines for applying protected area management categories including IUCN WCPA best practice guidance on recognising protected areas and assigning management categories and governance types* (IUCN, 2013).

S6. Land & Water Development Division, R., FAO. The World Digital Soil Map. Food and Agriculture Organization of the United Nations https://data.apps.fao.org/map/catalog/srv/eng/catalog.search#/home.

S7. Silva, J., Ribeiro, C., Guedes, R. *et al.* Roughness length classification of Corine Land Cover classes. In *Proceedings of the European wind energy conference, Milan, Italy*, vol. 710, 110 (2007).

S8. Hersbach, H. *et al.* The ERA5 global reanalysis. *Q. J. Royal Meteorol. Soc.* **146**, 1999–2049, https://doi.org/10.1002/qj.3803 (2020).

S9. Scholz, Y. Renewable energy based electricity supply at low costs: development of the REMix model and application for Europe (2012). Ph.D. thesis at Universität Stuttgart http://dx.doi.org/10.18419/opus-2015.

S10. Daniel Stetter. Enhancement of the REMix energy system model: Global renewable energy potentials, optimized power plant siting and scenario validation (2014). Ph.D. thesis at Universität Stuttgart http://dx.doi.org/10.18419/opus-6855.

S11. Portugal-Pereira, J., Soria, R., Rathmann, R., Schaeffer, R. & Szklo, A. Agricultural and agro-industrial residues-to-energy: Techno-economic and environmental assessment in Brazil. *Biomass Bioenergy* **81**, 521–533, https://doi.org/10.1016/j.biombioe.2015.08.010 (2015).

S12. Operador Nacional do Sistema Elétrico (ONS). Energia natural afluente por subsistema, http://www.ons.org.br/Paginas/resultados-da-operacao/historico-da-operacao/energia_afluente_subsistema.aspx (2021).

S13. Agência Nacional de Energia Elétrica (ANEEL). Sistema de Informação de Geração da ANEEL– SIGA, https://app.powerbi.com/view?r=eyJrIjoiNjc4OGYyYjQtYWM2ZC00YjllLWJlYmEtYzdkNTQ1MTc1NjM2IiwidCI6IjQwZDZmOWI4LWVjYTctNDZhMi05MmQ0LWVhNGU5YzAxNzBlMSIsImMiOjR9 (2021).

S14. Operador Nacional do Sistema Elétrico (ONS). Histórico da operação da curva de carga horária, http://www.ons.org.br/Paginas/resultados-da-operacao/historico-da-operacao/curva_carga_horaria.aspx (2021).

S15. Empresa de Pesquisa Energética (EPE). Anuário Estatístico de Energia Elétrica 2021, https://www.epe.gov.br/pt/publicacoes-dados-abertos/publicacoes/anuario-estatistico-de-energia-eletrica (2021).

S16. International Energy Agency (IEA). *World Energy Outlook 2021* (IEA, Paris, 2021). License: CC BY 4.0.

S17. Ministério de Minas e Energia & Empresa de Pesquisa Energética. Plano Nacional de Energia 2050, https://www.epe.gov.br/pt/publicacoes-dados-abertos/publicacoes/Plano-Nacional-de-Energia-2050 (2020).

S18. Baptista, L. B. *et al.* Good practice policies to bridge the emissions gap in key countries. *Glob. Environ. Chang.* **73**, 102472, https://doi.org/10.1016/j.gloenvcha.2022.102472 (2022).

S19. van Soest, H. L. *et al.* Global roll-out of comprehensive policy measures may aid in bridging emissions gap. *Nat. communications* **12**, 6419, https://doi.org/10.1038/s41467-021-26595-z (2021).

S20. Riahi, K. *et al.* Cost and attainability of meeting stringent climate targets without overshoot. *Nat. Clim. Chang.* **11**, 1063–1069, https://doi.org/10.1038/s41558-021-01215-2 (2021).

S21. Operador Nacional do Sistema Elétrico (ONS). Histórico da operação: Intercâmbios de energia, http://www.ons.org.br/Paginas/resultados-da-operacao/historico-da-operacao/intercambios_energia.aspx (2021).

S22. International Energy Agency. World energy model documentation, https://iea.blob.core.windows.net/assets/932ea201-0972-4231-8d81-356300e9fc43/WEM_Documentation_WEO2021.pdf (2021).

S23. Masson-Delmotte, V. *et al.* Summary for policymakers: Climate change 2021: The physical science basis. contribution of working group i to the sixth assessment report of the intergovernmental panel on climate change (2021). Cambridge University Press https://www.ipcc.ch/report/ar6/wg1/downloads/report/IPCC_AR6_WGI_SPM.pdf.
